# LncRNA Lnc‐APUE is Repressed by HNF4*α* and Promotes G1/S Phase Transition and Tumor Growth by Regulating MiR‐20b/E2F1 Axis

**DOI:** 10.1002/advs.202003094

**Published:** 2021-02-01

**Authors:** Song‐Yang Li, Ying Zhu, Ruo‐Nan Li, Jia‐Hui Huang, Kai You, Yun‐Fei Yuan, Shi‐Mei Zhuang

**Affiliations:** ^1^ MOE Key Laboratory of Gene Function and Regulation School of Life Sciences Collaborative Innovation Center for Cancer Medicine Sun Yat‐sen University Guangzhou 510275 China; ^2^ Department of Hepatobilliary Oncology Cancer Center Sun Yat‐sen University Guangzhou 510060 China

**Keywords:** cell cycle, ceRNA, hepatocellular carcinoma, lnc‐APUE, noncoding RNA

## Abstract

Many long noncoding RNAs (lncRNAs) have been annotated, but their functions remain unknown. The authors found a novel lnc‐APUE (lncRNA accelerating proliferation by upregulating E2F1) that is upregulated in different cancer types, including hepatocellular carcinoma (HCC), and high lnc‐APUE level is associated with short recurrence‐free survival (RFS) of HCC patients. Gain‐ and loss‐of‐function analyses showed that lnc‐APUE accelerated G1/S transition and tumor cell growth in vitro and allows hepatoma xenografts to grow faster in vivo. Mechanistically, lnc‐APUE binds to miR‐20b and relieves its repression on E2F1 expression, resulting in increased E2F1 level and accelerated G1/S phase transition and cell proliferation. Consistently, lnc‐APUE level is positively associated with the expression of E2F1 and its downstream target genes in HCC tissues. Further investigations disclose that hepatocyte nuclear factor 4 alpha (HNF4*α*) binds to the lnc‐APUE promoter, represses lnc‐APUE transcription, then diminishes E2F1 expression and cell proliferation. HNF4*α* expression is reduced in HCC tissues and low HNF4*α* level is correlated with high lnc‐APUE expression. Collectively, a HNF4*α*/lnc‐APUE/miR‐20b/E2F1 axis in which HNF4*α* represses lnc‐APUE expression and keeps E2F1 at a low level is identified. In tumor cells, HNF4*α* downregulation leads to lnc‐APUE upregulation, which prevents the inhibition of miR‐20b on E2F1 expression and thereby promotes cell cycle progression and tumor growth.

## Graphical abstract

There exists a HNF4*α*/lnc‐APUE/miR‐20b/E2F1 regulatory axis, in which HNF4*α* represses lnc‐APUE expression and keeps E2F1 at a low level. In tumor cells, HNF4*α* downregulation leads to lnc‐APUE upregulation, which may work as a miR‐20b sponge to prevent the miR‐20b‐mediated repression on E2F1 expression, resulting in increase of E2F1 level and in turn acceleration of G1/S transition and tumor growth.

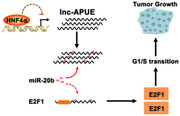

## 1 Introduction

Noncoding RNAs (ncRNAs) consist of small noncoding RNAs (<200 nucleotides) and long noncoding RNAs (lncRNAs, >200 nucleotides).^[^ ^1^ ^]^ MicroRNAs (miRNAs) are a class of transcripts with a length of ≈22 nucleotides, which repress gene expression by binding to the RNA sequence of target genes and thereby regulate various cell activities.^[^ ^2^ ^]^ Increasing evidences indicate that lncRNAs play critical roles in both physiological and pathological processes, and they may exert functions by binding to DNA, RNA, and proteins.^[^ ^3^, ^4^, ^5^ ^]^ Although thousands of lncRNAs have been annotated, the function and signaling networks of most lncRNAs remain unknown.

The transition from G1 to S phase is a key regulatory point in the cell cycle, and its misregulation contributes to unrestrained cell proliferation and consequent tumor development. The G1/S transition is tightly regulated by the retinoblastoma protein (pRb)‐E2F1 pathway, which primarily includes pRb, cyclins D and E, cyclin‐dependent kinase (CDK)4/6/2, CDK inhibitors, and E2F1.^[^ ^6^, ^7^ ^]^ Deregulation of the pRb‐E2F1 pathway is observed in various tumor and some anticancer drugs targeting the regulator of G1/S transition have achieved promising therapeutic effect.^[^ ^8^ ^]^ Therefore, identifying new regulators of G1/S transition and exploring their roles in tumor development may not only extend our understanding on the mechanisms of cell cycle control and tumorigenesis but also provide potential therapeutic targets for cancer therapy, which hold great biomedical significance.

Hepatocellular carcinoma (HCC) is a prevalent liver malignancy with rapid growth, early metastasis, and high mortality. Very limited drugs are available for HCC treatment.^[^ ^9^ ^]^ It is in urgent need to get deeper understanding on the mechanisms of HCC development and to identify new molecular targets for HCC therapy. Recently, lncRNAs emerge as regulators of G1/S transition and HCC development. We find that lncRNA lnc‐UCID binds to DEAH (Asp‐Glu‐Ala‐His) box helicase 9 (DHX9) and abolishes the function of DHX9 in decreasing CDK6 level, which promotes G1/S transition and HCC cell proliferation.^[^ ^10^ ^]^ LncRNA UFC1 accelerates G1/S transition and HCC cell proliferation in a human antigen R (HuR)/*β*‐catenin‐dependent manner.^[^ ^11^ ^]^ Linc00441 decreases pRb expression by recruiting DNA methyltransferase 3A (DNMT3A) to the promoter of pRb and thus enhancing CpG island methylation, resulting in HCC cell proliferation.^[^ ^12^ ^]^ Lnc‐HUR1 facilitates G1/S transition and HCC development by interacting with p53 to block the transcription of p53 downstream gene.^[^ ^13^ ^]^ MCM3AP‐AS1 drives G1/S transition and enhances HCC cell proliferation by targeting miR‐194/forkhead box A1 (FOXA1) axis.^[^ ^14^ ^]^ These findings imply that lncRNAs are important nodes in the regulatory network of cell cycle and proliferation. Obviously, more extensive investigations are required to find those lncRNAs that play critical roles in G1/S transition and HCC development.

The transcription factor hepatocyte nuclear factor 4 alpha (HNF4*α*) is highly expressed in the liver, and its downregulation is required for HCC development.^[^ ^15^, ^16^ ^]^ Herein, we identified a new oncogenic lncRNA that was upregulated in HCC and named it lnc‐APUE (lncRNA accelerating proliferation by upregulating E2F1). Lnc‐APUE transcription was repressed by HNF4*α*, and the downregulation of HNF4*α* resulted in upregulation of lnc‐APUE in HCC. Furthermore, lnc‐APUE accelerated G1/S phase transition and hepatoma cell growth by acting as a miR‐20b sponge to upregulate E2F1 expression. These findings identify a novel lnc‐APUE regulatory axis and disclose its biological function in cell cycle control and tumor development.

## 2 Results

### 2.1 Lnc‐APUE Is Elevated in HCC Tissues and Promotes Hepatoma Cell Growth In Vitro and In Vivo

**Figure 1:**
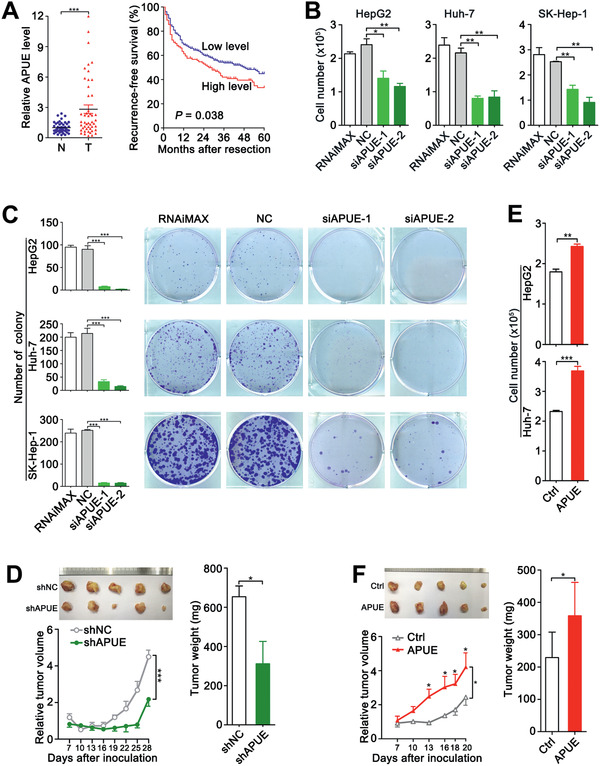
Lnc‐APUE is upregulated in HCC tissues and promotes in vitro and in vivo hepatoma cell growth. A) Upregulation of lnc‐APUE was associated with shorter recurrence‐free survival (RFS). Left panel, lnc‐APUE level was elevated in HCC tissues. Lnc‐APUE was detected by real‐time quantitative PCR (qPCR) in 51 paired HCC (T) and adjacent noncancerous liver tissues (N). The mean value of lnc‐APUE level in noncancerous livers was set as relative level 1. Right panel, a Kaplan‐Meier analysis showed that higher lnc‐APUE level was associated with shorter RFS. Using the minimum *p*‐value method, the 32th percentile of the lnc‐APUE level in 347 HCCs was selected as the cut‐off value to separate the lnc‐APUE‐high group (*n* = 110) from the lnc‐APUE‐low group (*n* = 237). B) Silencing lnc‐APUE repressed in vitro cell growth. C) Silencing lnc‐APUE inhibited colony formation. RNAiMAX: cells treated with Lipofectamine RNAiMAX without RNA duplexes. NC: cells transfected with negative control RNA duplex. siAPUE‐1 and siAPUE‐2: cells transfected with siRNA targeting different sequences of lnc‐APUE. D) Silencing lnc‐APUE suppressed xenograft growth in vivo. SK‐shAPUE and SK‐shNC stable cell lines were subcutaneously injected into NCG mice (*n* = 5 mice/group). E) Lnc‐APUE overexpression promoted hepatoma cell growth in vitro. F) Lnc‐APUE overexpression promoted xenograft growth in vivo. SK‐APUE and SK‐Ctrl stable cell lines were subcutaneously injected into NCG mice (*n* = 5 mice/group). For (D) and (F), the relative tumor volume and the weight and photographs of excised tumors are shown. For the relative tumor volume, values shown are fold change of tumor volume at indicated times relative to the mean volume of the control group in day 7. The data from at least three independent experiments are presented as mean ± SEM (A–C,E); *p*‐values were assessed by paired (A, *left*; D, F, *right*) or unpaired (B, C, E) Student′s *t*‐test, or log‐rank test (A, *right*) or two‐way ANOVA (D,F, *left*). *, *p* < 0.05; **, *p* < 0.01; ***, *p* < 0.001.

In an attempt to screen for oncogenic lncRNA, we performed a bioinformatic analysis based on two GEO datasets (GSE77314 and GSE115018) and found two candidate lncRNAs that fulfilled the following criteria (Figure S1A, Supporting Information): 1) more than twofold upregulation in HCC tissues compared to noncancerous livers in both datasets; 2) location in intergenic regions of human genome; 3) transcript number < 3. Subsequent gene ontology (GO) analysis revealed that lncRNA ENST00000515627 was highly co‐expressed with positive regulators of cell proliferation (Figure S1B, Supporting Information), and it was therefore selected for further investigation and we named it lnc‐APUE (lncRNA accelerating proliferation by upregulating E2F1) based on the functional analyses. Compared with noncancerous liver tissues, lnc‐APUE significantly increased in HCC tissues (**Figure** **1**A, left panel). Analysis on the transcriptome data from TCGA revealed frequent upregulation of lnc‐APUE in different cancer types (Figure S1C, Supporting Information). Furthermore, the Kaplan–Meier analysis revealed a correlation between high lnc‐APUE level in HCC tissues and short recurrence‐free survival (RFS) of patients (Figure 1A, right panel). Both univariate and multivariate analysis verified upregulation of lnc‐APUE as an independent prognostic factor for shorter RFS (Table S1, Supporting Information). We then characterized lnc‐APUE as an 1123‐nt polyadenylated RNA (Figure S2A,B, Supporting Information) that was located on chromosome 5 and had no protein‐coding potential (Figure S2C, Supporting Information).

We then examined whether lnc‐APUE affected cell growth, using three human hepatoma cell lines (HepG2, Huh‐7, and SK‐Hep‐1). HepG2, Huh‐7, and SK‐Hep‐1 cells were used in loss‐of‐function analyses, while HepG2 and Huh‐7 were employed for gain‐of‐function studies, and SK‐Hep‐1 was used in mouse xenograft models. Compared with NC‐transfected hepatoma cells, siAPUE‐transfectants (Figure S3A, Supporting Information) displayed reduced cell number (Figure 1B) and fewer and smaller colonies (Figure 1C). Analyses using mouse xenograft models revealed that knockdown of lnc‐APUE (Figure S3B, Supporting Information) significantly inhibited tumor growth in vivo (Figure 1D). Consistently, lnc‐APUE overexpression (Figure S3C, Supporting Information) increased cell number (Figure 1E) and promoted xenograft growth (Figure 1F). These findings suggest that lnc‐APUE may function as an oncogenic lncRNA to promote hepatoma cell growth in vitro and in vivo.

### 2.2 Lnc‐APUE Facilitates G1/S Phase Transition by Increasing E2F1 Level

**Figure 2:**
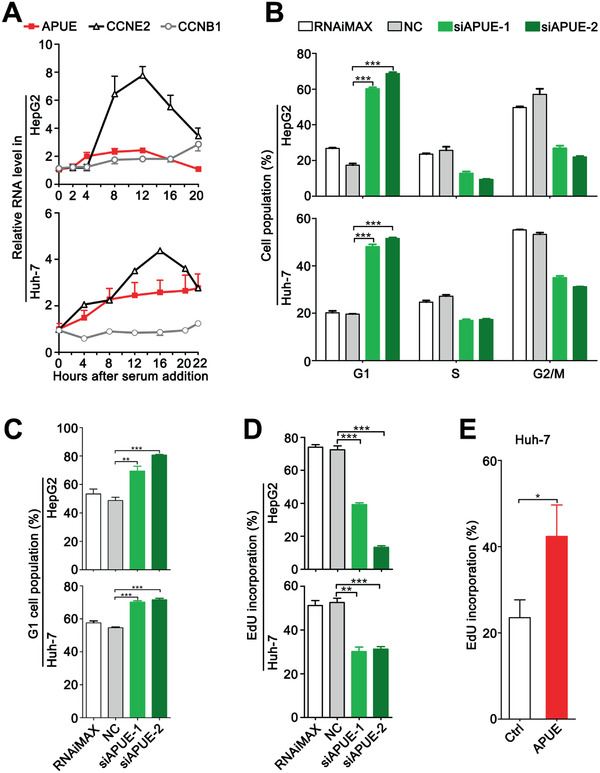
Lnc‐APUE promotes G1/S transition. A) The expression pattern of lnc‐APUE during cell cycle progression was examined using serum deprivation‐stimulation model. Serum‐deprived HepG2 and Huh‐7 cells were incubated with fresh medium containing serum for the indicated times. B,C) Silencing lnc‐APUE significantly increased the fraction of G1‐phase cells. Cells transfected with the indicated siRNAs were synchronized by B) nocodazole for 12 h, followed by fluorescence‐activated cell sorting (FACS) analysis, or C) serum‐starved for 48 h, then refreshed with medium containing serum and further incubated for 18(HepG2) or 20 h (Huh‐7) before FACS analysis. D) The fraction of DNA‐replicating cells was reduced by silencing lnc‐APUE. NC‐ or siAPUE‐transfectants were subjected to ethynyldeoxyuridine (EdU) incorporation assay. E) The fraction of DNA‐replicating cells was increased by overexpressing lnc‐APUE. Huh‐7‐APUE and Huh‐7‐Ctrl cells were subjected to EdU incorporation assay. The data from at least three independent experiments are presented as mean ± SEM (A–E); *p*‐values were assessed by unpaired Student′s *t*‐test (B–E). *, *p* < 0.05; **, *p* < 0.01; ***, *p* < 0.001.

To evaluate whether lnc‐APUE promoted cell growth by regulating cell cycle, the expression pattern of lnc‐APUE during cell cycle progression was first examined. It's well known that CCNE2 expression is induced at late G1 phase and declines after the entry of S phase, and CCNB1 starts to accumulate at late S phase. As shown, lnc‐APUE expression began to increase before CCNE2 elevation and remained at high level through late G1 phase, implying that lnc‐APUE may regulate G1/S transition (**Figure** **2**A). Subsequent analysis using nocodazole‐synchronized model revealed that silencing lnc‐APUE caused a significant accumulation of the G1‐population (Figure 2B). Serum starvation‐stimulation experiments further showed that compared to NC‐transfectants, much more siAPUE‐transfected cells stayed at the G1‐phase after serum re‐addition (Figure 2C). Consistently, the fraction of cells with DNA replication was reduced by silencing lnc‐APUE (Figure 2D) but increased by overexpressing lnc‐APUE (Figure 2E), suggesting that lnc‐APUE may accelerate G1/S phase transition and in turn promote cell proliferation.

**Figure 3:**
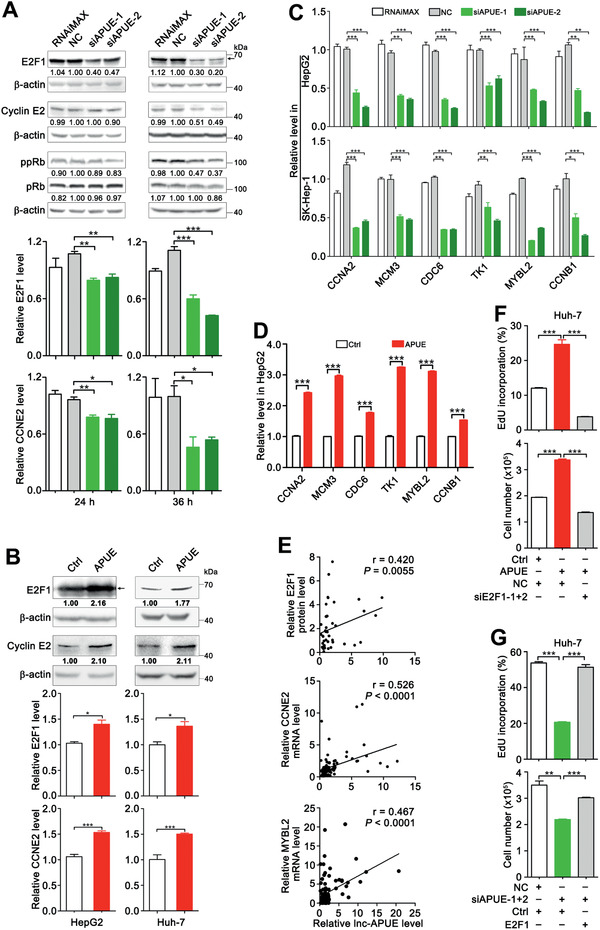
Lnc‐APUE accelerates G1/S phase transition by increasing E2F1 level. A) Lnc‐APUE knockdown decreased the protein and mRNA levels of E2F1 and CCNE2. HepG2 cells were transfected with NC or siAPUE for 24 or 36 h before immunoblotting and qPCR. B) Lnc‐APUE overexpression elevated the protein and mRNA levels of E2F1 and CCNE2. HepG2‐APUE/Huh‐7‐APUE stable cell lines and their control cell lines HepG2‐Ctrl/Huh‐7‐Ctrl were subjected to immunoblotting and qPCR. For (A,B), the arrow indicates the band for E2F1 protein; the level of target protein relative to *β*‐actin level is indicated under each band. C) Silencing lnc‐APUE decreased the levels of E2F1‐transactivated S phase genes. The mRNA levels were assessed by qPCR analysis at 36 h post‐transfection. D) Overexpressing lnc‐APUE promoted the expression of E2F1‐transactivated S phase genes. The mRNA levels were assessed by qPCR analysis in HepG2‐APUE and HepG2‐Ctrl cells. E) Significant correlation between upregulation of lnc‐APUE and elevation of E2F1 and CCNE2/MYBL2 in human HCC tissues. Lnc‐APUE and mRNAs of CCNE2 and MYBL2 were examined in 22 paired HCC tissues and noncancerous livers by qPCR, whereas E2F1 was detected by immunoblotting, as presented in Figure S5, Supporting Information. Spearman's correlation coefficient analysis was performed. F) Silencing E2F1 abrogated the effect of lnc‐APUE in increasing the number of DNA‐replicating cells and total cells. Huh‐7‐APUE and Huh‐7‐Ctrl were transfected with NC or with both siE2F1‐1 and siE2F1‐2. G) E2F1 expression antagonized the effect of siAPUE in decreasing the number of DNA‐replicating cells and total cells. Huh‐7‐E2F1 and Huh‐7‐Ctrl were transfected with NC or with both siAPUE‐1 and siAPUE‐2. For (F–G), “+” or “−”, presence (+) or absence (−) of the treatment. For (A–D,F,G), the data from at least three independent experiments are presented as mean ± SEM; *p*‐values were assessed by unpaired Student′s *t*‐test. *, *p* < 0.05; **, *p* < 0.01; ***, *p* < 0.001.

We further explored the molecular mechanisms whereby lnc‐APUE exerted its function. The levels of the central regulators of G1/S phase transition, including cyclin D1, cyclin E1/2, CDK4, CDK6, CDK2, p15, p16, pRb, phosphorylated pRb (ppRb), and E2F1 were examined at 24 and 36 h after silencing lnc‐APUE. As shown, reduction in the protein level of E2F1 was observed at 24 h and became more prominent at 36 h, whereas the protein levels of cyclin E2 and ppRb only decreased at 36 h after lnc‐APUE knockdown (**Figure** **3**A, upper panel). And the levels of other examined proteins remained unchanged at both time points (Figure S4, Supporting Information). It is known that E2F1 works as a transcription factor to induce transcription of CCNE2 and itself, and then the CCNE2‐encoded cyclin E2 phosphorylates pRb. We found that the mRNA levels of E2F1 and CCNE2 displayed time‐dependent reduction after lnc‐APUE silencing (Figure 3A, middle and lower panel). Consistently, overexpression of lnc‐APUE increased the protein levels (Figure 3B, upper panel) and the mRNA levels of E2F1 and CCNE2 (Figure 3B, middle and lower panel), suggesting that the observed alterations of CCNE2 and ppRb may result from change of E2F1 protein. We thus focused on the role of lnc‐APUE in regulating E2F1. As expected, the mRNA levels of S phase genes transactivated by E2F1 were reduced by lnc‐APUE silencing (Figure 3C) but were increased by lnc‐APUE overexpression (Figure 3D). And lnc‐APUE level was positively correlated with the protein level of E2F1 and the mRNA levels of E2F1 target genes in human HCC tissues, respectively (Figure 3E; Figure S5, Supporting Information). Moreover, silencing E2F1 abrogated the lnc‐APUE‐stimulated G1/S transition and cell growth (Figure 3F), whereas E2F1 overexpression (Figure S6, Supporting Information) antagonized the siAPUE‐induced blocking of G1/S transition and cell growth (Figure 3G). These data indicate that lnc‐APUE may accelerate G1/S phase transition and cell proliferation by increasing E2F1 level.

### 2.3 Lnc‐APUE Upregulates E2F1 Expression by Acting as a MiR‐20b Sponge

**Figure 4:**
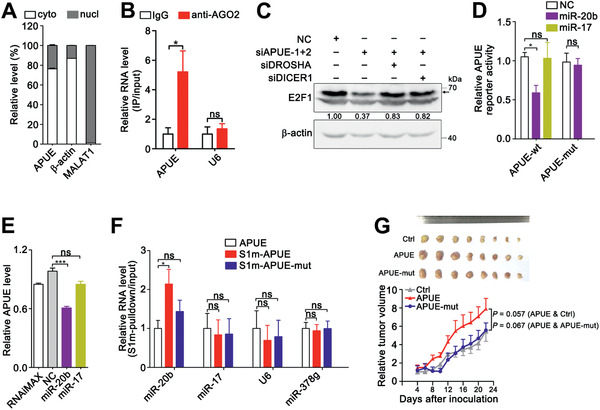
miR‐20b inhibits lnc‐APUE expression via a direct interaction. A) Lnc‐APUE is mainly localized in the cytoplasm. The RNA levels of lnc‐APUE, *β*‐actin, and MALAT1 in the subcellular fractions of HepG2 cells were detected by qPCR. Cyto, cytoplasm; nucl, nucleus. *β*‐actin and MALAT1 were used as positive controls for the fractions of cytoplasm and nucleus, respectively. B) Lnc‐APUE was associated with AGO2 in vivo. HepG2 cells were applied to RNA immunoprecipitation (RIP) analysis with an antibody against AGO2 or an isotype‐matched IgG (negative control), and the lnc‐APUE and U6 levels in the precipitates were analyzed by qPCR. C) Knockdown of DROSHA or DICER1 reversed the siAPUE‐induced suppression of E2F1 expression. HepG2 cells were co‐transfected with siRNA duplexes for 36 h. “+” or “−”, with (+) or without (−) the indicated treatment. The arrow indicates the band for E2F1 protein; the level of E2F1 relative to *β*‐actin level is indicated under each band. D) miR‐20b overexpression reduced the activity of firefly luciferase with the wild‐type miR‐20b‐binding sequences of lnc‐APUE. HepG2 cells were co‐transfected with NC, miR‐20b, or miR‐17 mimics, and the luciferase reporter plasmids that carried either wild‐type (wt) or mutant (mut) miR‐20b‐binding sequences of lnc‐APUE for 48 h, which is then subjected to luciferase activity assay. E) miR‐20b overexpression suppressed the expression of lnc‐APUE. HepG2 cells were transfected with NC, miR‐20b, or miR‐17 for 48 h, and then subjected to qPCR analysis. F) Lnc‐APUE physically associated with cellular miR‐20b. The HepG2‐APUE, HepG2‐S1m‐APUE, and HepG2‐S1m‐APUE‐mut stable cell lines were applied to RNA affinity purification. The indicated RNAs in the S1m‐pulldown precipitates and in the input were detected by qPCR. The RNA level in the pulldown product was corrected by that in the input. U6 and miR‐378g: negative controls. G) Mutation of the miR‐20b‐binding sites in lnc‐APUE abolished the lnc‐APUE's tumor‐promoting effect. SK‐Ctrl, SK‐APUE, and SK‐APUE‐mut stable cell lines were subcutaneously injected into NCG mice (*n* = 8 mice/group). The relative tumor volume and the photographs of excised tumors are shown. For the relative tumor volume, values shown are fold change of tumor volume at the indicated times relative to the mean volume of the control group at day 4. The data from at least three independent experiments are presented as mean ± SEM (A,B,D–F); *p*‐values were assessed by unpaired Student′s *t*‐test (A,B,D–F) or two‐way ANOVA (G). *, *p* < 0.05; ***, *p* < 0.001; ns, not significant.

We next explored how lnc‐APUE increased E2F1 expression. As shown, lnc‐APUE was predominantly localized in the cytoplasm (**Figure** **4**A). We thus examined whether lnc‐APUE acted as a miRNA sponge. RNA immunoprecipitation (RIP) experiment was conducted using anti‐AGO2 antibody, a key component that associates with miRNA. The results showed that lnc‐APUE was present in the AGO2‐precipitates, while the negative control U6 was undetectable in the precipitates (Figure 4B). Moreover, knockdown of DROSHA or DICER1, the key regulators for miRNA biogenesis, abolished the siAPUE‐induced downregulation of E2F1 (Figure 4C). These data indicate that lnc‐APUE may increase E2F1 expression via regulating miRNA.

Subsequent analysis using TargetScan and RegRNA prediction algorithm identified potential binding sites of four miRNAs on both lnc‐APUE and E2F1‐3’UTR. Among them, miR‐4459 and miR‐4722 were excluded due to very low expression. miR‐20b and miR‐17, each of which had two putative binding sites on both lnc‐APUE and E2F1‐3’UTR (Figure S7A,B, Supporting Information), were selected for further study. Dual‐luciferase reporter analysis showed that overexpression of miR‐20b, but not miR‐17, suppressed the activity of luciferase containing full‐length lnc‐APUE, and this effect was abated if the miR‐20b‐binding site in lnc‐APUE was mutated (Figure 4D). Consistently, overexpression of miR‐20b, but not miR‐17, reduced the level of cellular lnc‐APUE (Figure 4E). To verify the direct interaction between miR‐20b and lnc‐APUE, we applied RNA affinity purification to pull down cellular miR‐20b that bound to lnc‐APUE. The full‐length lnc‐APUE containing wild‐type or mutant miR‐20b‐binding sites was tagged with S1m, a streptavidin‐binding RNA aptamer (Figure S8A, Supporting Information). HepG2 cells that stably expressed S1m‐APUE, S1m‐APUE‐mut or untagged APUE were applied to RNA affinity purification with streptavidin beads. As shown, lnc‐APUE was enriched in the precipitates from S1m‐APUE‐ and S1m‐APUE‐mut‐transfectants, compared to the precipitates from untagged‐APUE‐transfectanted cells (Figure S8B, Supporting Information). Compared to the untagged‐APUE transfectants, miR‐20b was dramatically enriched in the S1m‐APUE‐precipitates but not in the S1m‐APUE‐mut‐precipitates, and no enrichment of miR‐17 or the negative controls (U6 and miR‐378g) was observed in any precipitates (Figure 4F). Moreover, xenograft growth was promoted by overexpressing wild‐type lnc‐APUE, but was not affected by overexpressing lnc‐APUE with mutation in the miR‐20b‐binding sites (Figure 1F; Figure 4G). These findings indicate that lnc‐APUE may physically bind to miR‐20b in vivo and this interaction is critical for the tumor‐promoting effect of lnc‐APUE.

**Figure 5:**
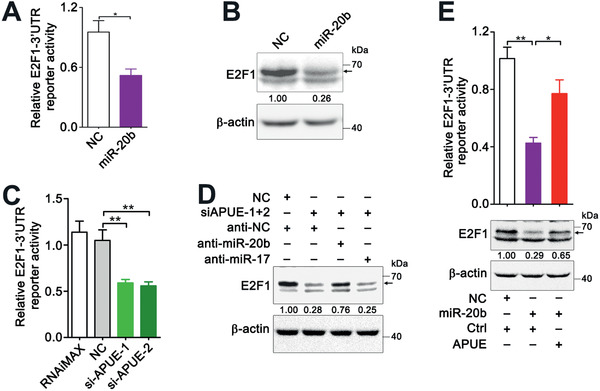
Lnc‐APUE relieves the repression of miR‐20b on E2F1 expression. A) miR‐20b reduced the activity of firefly luciferase carrying the miR‐20b‐binding sequences of E2F1‐3’UTR. HepG2 cells were co‐transfected with NC or miR‐20b mimics and the luciferase reporter plasmids carrying the miR‐20b‐binding sequences of E2F1‐3’UTR for 48 h, then subjected to luciferase activity assay. B) miR‐20b overexpression decreased the level of E2F1 protein. HepG2 cells were transfected with RNA duplexes for 48 h before immunoblotting. C) Silencing lnc‐APUE reduced the activity of E2F1‐3’UTR reporter. NC‐ or siAPUE‐transfectants were transfected with luciferase reporter plasmid carrying the miR‐20b‐binding sequences of E2F1‐3’UTR for 48 h, then subjected to luciferase activity assay. D) Antagonism of miR‐20b reversed the siAPUE‐induced suppression in E2F1 expression. HepG2 cells were co‐transfected with the indicated siRNA duplex and miRNA inhibitor for 36 h, and then subjected to immunoblotting. E) Overexpression of lnc‐APUE abated the function of miR‐20b in reducing the activity of E2F1‐3’UTR reporter and the level of E2F1 protein. HepG2‐APUE and HepG2‐Ctrl were co‐transfected with the indicated RNA duplexes and luciferase reporter plasmid carrying the miR‐20b‐binding sequences of E2F1‐3’UTR for 48 h, then subjected to luciferase activity assay (upper panel). HepG2‐APUE and HepG2‐Ctrl were transfected with the indicated RNA duplexes for 48 h before immunoblotting (lower panel). For (D,E), “+” or “−” indicates presence (+) or absence (−) of the indicated treatment. For (B,D,E), arrow indicates the band for E2F1; the level of E2F1 relative to *β*‐actin level is indicated under each band. For (A,C,E), the data from at least three independent experiments are presented as mean ± SEM; *p*‐values were assessed by unpaired Student′s *t*‐test. *, *p* < 0.05; **, *p* < 0.01.

We then examined whether lnc‐APUE upregulated E2F1 expression by acting as a miR‐20b sponge. We found that overexpression of miR‐20b significantly reduced the activity of firefly luciferase carrying E2F1‐3’UTR (**Figure** **5**A) and reduced the E2F1 protein level (Figure 5B). Furthermore, silencing lnc‐APUE attenuated the activity of luciferase with E2F1‐3’UTR (Figure 5C), which mimicked the effect of miR‐20b overexpression. Moreover, the effect of siAPUE in reducing E2F1 protein was abolished by miR‐20b inhibitor but was not affected by anti‐miR‐17 (Figure 5D). Consistently, overexpressing lnc‐APUE abated the role of miR‐20b in inhibiting the activity of luciferase with E2F1‐3’UTR and in reducing the level of cellular E2F1 protein (Figure 5E). Taken together, lnc‐APUE may upregulate E2F1 by binding to miR‐20b and attenuating its repression on E2F1 expression.

### 2.4 Downregulation of HNF4*α* Is Correlated with Upregulation of Lnc‐APUE in Tumor Tissues

**Figure 6:**
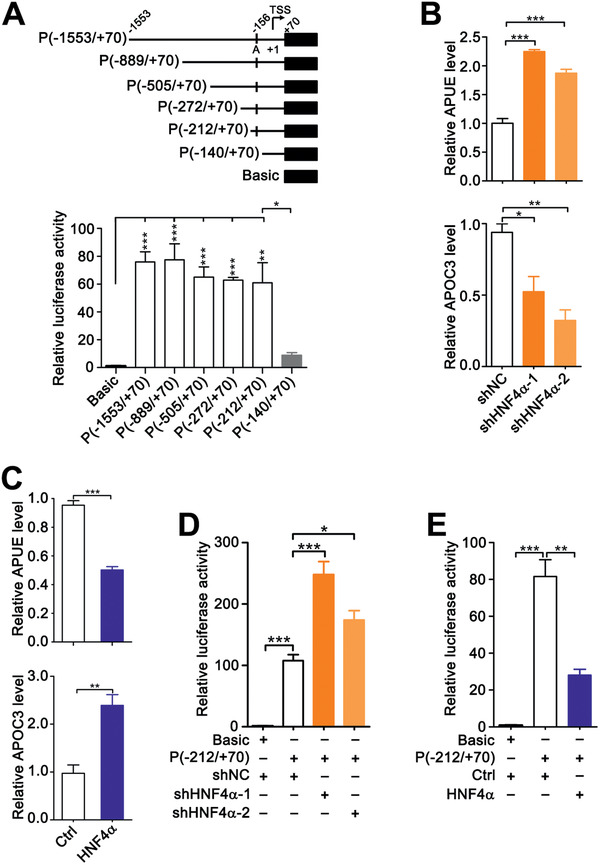
HNF4*α* suppresses lnc‐APUE transcription. A) Characterization of the lnc‐APUE promoter by 5’‐deletion analysis. Upper panel, schematic diagram of firefly luciferase reporters carrying the indicated DNA fragments upstream of lnc‐APUE. Arrow designates the transcription direction of lnc‐APUE. Putative HNF4*α* binding site is depicted as short vertical line (denoted as A). TSS: transcription start site. Lower panel, luciferase reporter assays. HepG2 cells were co‐transfected with pRL‐TK and the indicated plasmids for 48 h, and then subjected to luciferase activity assay. B) Knockdown of HNF4*α* increased lnc‐APUE level. The levels of lnc‐APUE and APOC3 in HepG2‐shHNF4*α* and its control line HepG2‐shNC were examined by qPCR analysis. APOC3, positive control. C) HNF4*α* overexpression reduced lnc‐APUE level. The expression of lnc‐APUE and APOC3 was examined in HepG2‐HNF4*α* and its control line HepG2‐Ctrl by qPCR analysis. D) Knockdown of HNF4*α* enhanced the lnc‐APUE promoter activity. HepG2‐shHNF4*α* and HepG2‐shNC lines were transfected with the indicated vectors for 48 h, followed by luciferase activity assay. E) Overexpression of HNF4*α* reduced the lnc‐APUE promoter activity. HepG2‐HNF4*α* and HepG2‐Ctrl lines were transfected with the indicated vectors for 48 h, and then subjected to luciferase activity assay. For (A–E), the data from at least three independent experiments are presented as mean ± SEM; *p*‐values were assessed by unpaired Student′s *t*‐test. *, *p* < 0.05; **, *p* < 0.01; ***, *p* < 0.001.

We further elucidated the mechanism underlying lnc‐APUE upregulation in HCC. Lnc‐APUE was located on chromosome 5q31.1, an intergenic fragment that didn't show prevalent amplification in HCC. Therefore, deregulated transcription rather than genome amplification may account for lnc‐APUE upregulation. To examine this hypothesis, we first mapped the lnc‐APUE promoter. Chromatin immunoprecipitation (ChIP)‐sequencing data from ENCODE revealed that H3K4Me3, H3K27Ac, and clusters of DNase I hypersensitive sites were enriched in the 1.5‐kb region upstream of the transcriptional start site of lnc‐APUE (Figure S9A, Supporting Information), indicating the existence of an active promoter. A luciferase reporter construct P(−1553/+70), which carried the ≈−1553–+70‐bp fragment of lnc‐APUE, exhibited much higher activity than the control plasmid pGL3‐basic, suggesting that this segment may contain the lnc‐APUE promoter (**Figure** **6**A). To further validate the core promoter region, a 5’‐deletion analysis was performed. As shown, deleting the ≈−1553–−212‐bp region of lnc‐APUE did not affect the activity of the luciferase reporter (Figure 6A). However, the reporter construct P(−140/+70), which only contained the ≈−140–+70‐bp sequence of lnc‐APUE, showed very low luciferase activity (Figure 6A), suggesting the ≈−212–−140‐bp region as the core promoter of lnc‐APUE.

**Figure 7:**
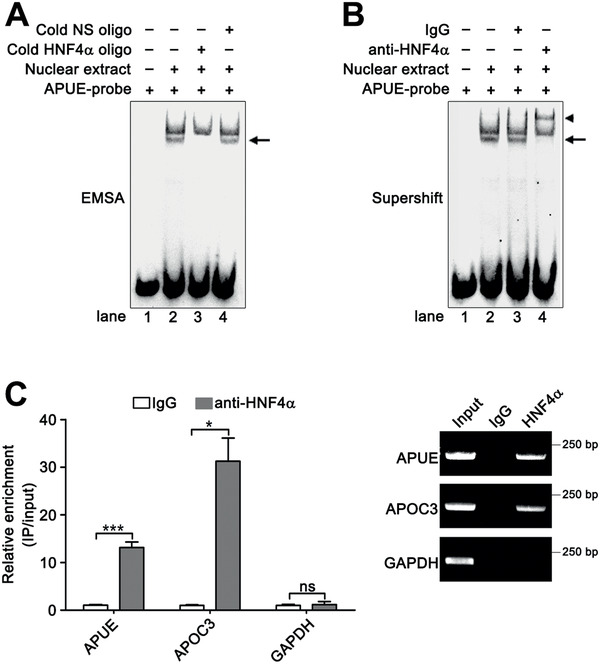
HNF4*α* binds to the lnc‐APUE promoter in vitro and in vivo. A,B) HNF4*α* interacted with the predicted HNF4*α*‐binding sequence in the lnc‐APUE promoter. A) EMSA and B) antibody‐supershift assay were used. Nuclear proteins were obtained from HepG2 cells. APUE‐probe: a biotin‐labeled probe carrying the predicted HNF4*α*‐binding site at the ≈−156–−141‐bp region of the lnc‐APUE promoter. Cold NS oligo: a nonspecific oligonucleotide without biotin label. Cold HNF4*α* oligo: a HNF4*α* consensus binding sequence without biotin label. The DNA‐protein complexes are denoted by arrow and the supershift band are denoted by arrow head. “+” or “−” indicates presence (+) or absence (−) of the indicated treatment. C) ChIP analysis showed a direct interaction between HNF4*α* and the lnc‐APUE promoter in vivo. HepG2 cells were subjected to ChIP analysis using anti‐HNF4*α* or isotype‐matched control IgG, and the amount of the lnc‐APUE promoter were determined by qPCR analysis (left panel) and semi‐quantitative PCR assay (right panel). The promoters of GAPDH and APOC3 were used as a negative and positive control, respectively. For (C), the data from at least three independent experiments are presented as mean ± SEM; *p*‐values were assessed by unpaired Student′s *t*‐test. *, *p* < 0.05; ***, *p* < 0.001; ns, not significant.

Bioinformatics analyses identified putative binding sites of HNF4*α*, hepatocyte nuclear factor 4 gamma (HNF4G), and retinoid X receptor alpha (RXRA) within the core promoter of lnc‐APUE (Figure S9B,C, Supporting Information). Silencing of HNF4*α*, but not HNF4G or RXRA, increased the level of cellular lnc‐APUE (Figure 6B; Figure S9D,E, Supporting Information), while HNF4*α* overexpression inhibited lnc‐APUE expression (Figure 6C). Notably, the mRNA level of apolipoprotein C‐III (APOC3), which is transactivated by HNF4*α*,^[^ ^17^ ^]^ was decreased by HNF4*α* silencing but was increased by HNF4*α* overexpression (Figure 6B,C). Furthermore, the promoter activity of P(−212/+70) was increased by silencing HNF4*α* (Figure 6D) but was reduced by overexpressing HNF4*α* (Figure 6E), suggesting that HNF4*α* may repress the transcription of lnc‐APUE. Subsequent electrophoretic mobility shift assay (EMSA) (**Figure** **7**A) and antibody‐supershift analysis (Figure 7B) revealed that HNF4*α* interacted with the core promoter sequence of lnc‐APUE in vitro. Moreover, ChIP assays disclosed that HNF4*α* interacted with the lnc‐APUE promoter in vivo (Figure 7C). These findings imply that HNF4*α* may inhibit lnc‐APUE transcription by directly interacting with the lnc‐APUE promoter.

**Figure 8:**
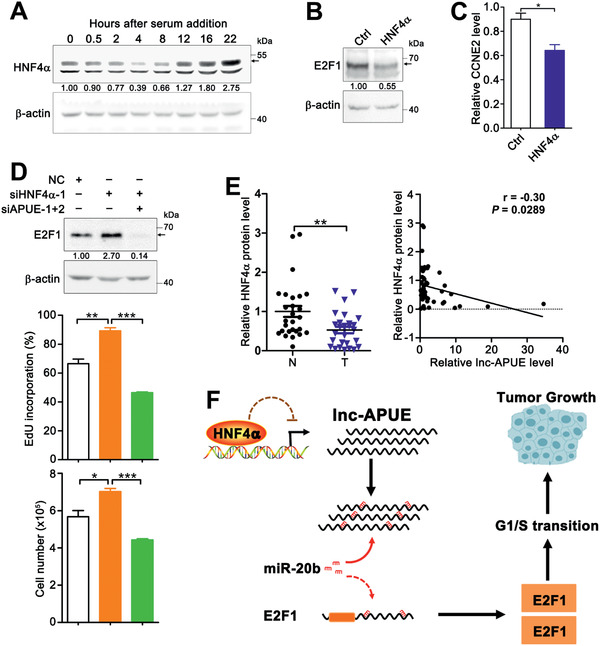
HNF4*α* inhibits E2F1 expression by regulating lnc‐APUE expression. A) The expression of HNF4*α* began to reduce once cells re‐entered a cell cycle. HepG2 cells were serum‐starved for 48 h, followed by incubation in a serum‐containing medium for different times. B,C) Overexpression of HNF4*α* decreased the protein level of E2F1 and the mRNA level of CCNE2. HepG2‐HNF4*α* and HepG2‐Ctrl cells were subjected to B) immunoblotting and C) qPCR analysis. D) Silencing lnc‐APUE abrogated the siHNF4*α*‐induced elevation in the level of E2F1 protein and the number of DNA‐replicating cells and total cells. HepG2 cells were transfected with the indicated RNA duplexes before being subjected to immunoblotting (top panel), EdU incorporation (middle panel), and cell counting (bottom panel) assays. E) The HNF4*α* protein level was reduced in HCC tissues and inversely correlated with lnc‐APUE level. HNF4*α* was assessed in 27 paired HCC tissues (T) and noncancerous livers (N) by immunoblotting, as presented in Figure S10, Supporting Information. For right panel, Spearman's correlation coefficient analysis was performed. F) The model of the HNF4*α*/lnc‐APUE/miR‐20b/E2F1 regulatory axis and its function in G1/S transition and tumor growth. For (A,B,D), the band for HNF4*α* or E2F1 is indicated by arrow; the level of target protein relative to *β*‐actin level is indicated under each band. For (C,D), the data from at least three independent experiments are presented as mean ± SEM; *p*‐values were assessed by unpaired Student′s *t*‐test. *, *p* < 0.05; **, *p* < 0.01; ***, *p* < 0.001.

We thereby explored whether HNF4*α* could inhibit E2F1 expression by regulating lnc‐APUE. Serum starvation‐stimulation assays revealed that the expression of HNF4*α* began to reduce once cells re‐entered a cell cycle, and displayed the lowest level at 4 h after serum re‐addition (**Figure** **8**A), when the levels of lnc‐APUE and CCNE2 started to increase (Figure 2A). Consistently, HNF4*α* overexpression decreased the protein level of E2F1 (Figure 8B) and the mRNA level of CCNE2 (Figure 8C), which mimicked the effects of lnc‐APUE knockdown (Figure 3A). And inhibition of lnc‐APUE expression abrogated the stimulatory effect of silencing HNF4*α* on E2F1 expression, G1/S transition, and cell growth (Figure 8D). Moreover, the protein level of HNF4*α* was frequently downregulated and had a negative correlation with lnc‐APUE expression in HCC tissues (Figure 8E; Figure S10, Supporting Information).

Taken together, downregulation of HNF4*α* may lead to the upregulation of lnc‐APUE, and lnc‐APUE may work as a miR‐20b sponge to prevent the miR‐20b‐mediated repression on E2F1 expression, thereby promoting G1/S transition and tumor growth (Figure 8F).

## 3 Discussion

The misregulation of cell cycle, especially deficiency in the control of G1/S transition, is a key event in tumor development, and cell cycle regulators are therefore considered as attractive targets in cancer therapy.^[^ ^8^ ^]^ Apart from miRNAs and proteins, lncRNAs emerge as important players in different cell activities. In this study, we find a novel oncogenic lncRNA lnc‐APUE and elucidate its biological function and implication in HCC development. HNF4*α*, a tumor suppressor of HCC development, directly represses lnc‐APUE transcription. Downregulation of HNF4*α* in HCC results in upregulation of lnc‐APUE, leading to enhanced E2F1 expression and in turn accelerating G1/S transition and tumor growth.

E2F1 works as a transcription factor that regulates cell cycle by transactivating multiple genes that are required for G1/S transition and DNA replication.^[^ ^7^ ^]^ The transactivation capacity of E2F1 is dependent on its binding partners, like dimerization proteins (DPs) and pRb.^[^ ^18^ ^]^ In quiescent state, hypophosphorylated pRb bound to E2F1, masks the transcriptional activation domain of the E2F1‐DP heterodimer and blocks the binding of transcriptional co‐activators to target genes’ promoters.^[^ ^18^, ^19^ ^]^ Upon stimulation of growth signals, pRb is phosphorylated and then releases E2F, which in turn induces a transcription of S phase genes. The level of E2F1 is controlled by transcription factors, such as kuppel like factor 6 (KLF6),^[^ ^20^ ^]^ E2F1,^[^ ^21^ ^]^ E2F7, and E2F8,^[^ ^22^ ^]^ by the ubiquitin‐proteasome system including Skp1‐Cul1‐F box (SCF)‐cyclin F^[^ ^23^ ^]^ and POH1,^[^ ^24^ ^]^ and by miRNAs, like miR‐183,^[^ ^25^ ^]^ miR‐17, and miR‐20a/b.^[^ ^26^ ^]^ Recent evidence indicates that lncRNA may also regulate E2F1 expression. It has been shown that EMS (E2F1 mRNA stabilizing factor) interacts with the RNA binding protein RALY to enhance E2F1 mRNA stability.^[^ ^27^ ^]^ Here, we identified a novel lncRNA lnc‐APUE that upregulated E2F1 expression by binding to miR‐20b and relieved the repression of miR‐20b on E2F1 expression based on following evidences: 1) lnc‐APUE was predominantly localized in the cytoplasm and associated with AGO2. 2) Bioinformatics analysis, luciferase reporter assays, RNA affinity purification experiment, and biochemical analyses revealed that miR‐20b bound to lnc‐APUE and E2F1‐3’UTR, and repressed their expression. 3) Both gain‐ and loss‐of function studies revealed that lnc‐APUE abolished the function of miR‐20b in reducing the protein level of E2F1.

miR‐20b is upregulated in multiple types of cancer, including HCC.^[^ ^28^ ^]^ However, whether miR‐20b has growth stimulatory or inhibitory activity remains unclear. miR‐20b can decrease the levels of tumor suppressors, like phosphatase and tensin homolog (PTEN),^[^ ^29^ ^]^ but it also suppresses the expression of oncogenes, such as cyclin D1^[^ ^30^ ^]^ and CDK6.^[^ ^31^ ^]^ We found that miR‐20b inhibited E2F1 expression and lnc‐APUE had no impact on the levels of cyclin D1 and CDK6 in our cell models. Furthermore, the effect of lnc‐APUE in promoting xenograft growth was abrogated when the miR‐20b‐binding sites in lnc‐APUE were mutated. Hence, lnc‐APUE upregulation may promote HCC development by binding to miR‐20b and specifically abrogating the suppression of miR‐20b on E2F1 expression.

HNF4*α* is a transcription factor that belongs to the nuclear receptor superfamily and is enriched in the liver, pancreas, gastrointestinal tract, and kidneys.^[^ ^32^ ^]^ HNF4*α* is essential for liver function owing to its critical role in regulating the expression of genes, such as apolipoproteins, blood coagulation factors, and enzymes involved in glycolysis, ureagenesis, gluconeogenesis, synthesis of apolipoprotein or bile acid, metabolism of a fatty acid or drug.^[^ ^15^ ^]^ Disruption of HNF4*α* function has been observed in various liver diseases, like metabolic syndrome,^[^ ^33^ ^]^ type 2 diabetes,^[^ ^34^ ^]^ and HCC.^[^ ^15^ ^]^ HNF4*α* has the capacity to suppress hepatocyte proliferation and hepatocarcinogenesis.^[^ ^15^ ^]^ The rapid decline of HNF4*α* protein levels resulted in sustained hepatocyte cell proliferation accompanied by enhancing c‐Myc and cyclin D1 expression after 2/3 partial hepatectomy.^[^ ^35^ ^]^ Downregulation of HNF4*α* is associated with HCC progression in rodents and humans.^[^ ^15^, ^17^ ^]^ HNF4*α* is mostly known to activate transcription, but it can also suppress transcription^[^ ^36^ ^]^ depending on its interactions with coactivator (Glutamate receptor interacting protein 1 (GRIP1), SRC‐1, CREB binding protein (CBP)/E1A binding protein p300 (p300))^[^ ^37^, ^38^, ^39^ ^]^ or corepressor silencing mediator of retinoid and thyroid receptors (SMRT).^[^ ^40^ ^]^ Previous studies mainly focused on the transcriptional regulation of mRNA and miRNA and there are no reports on the HNF4*α*‐mediated repression on lncRNAs expression yet. In this study, we identified the promoter region of lnc‐APUE, verified that HNF4*α* directly interacted with the lnc‐APUE promoter to repress its transcription, and demonstrated that HNF4*α* downregulation represented an important mechanism responsible for lnc‐APUE upregulation in HCC.

LncRNAs usually exhibit poor sequence conservation across species.^[^ ^5^, ^41^ ^]^ Analyses on the conservation of lnc‐APUE locus across multi‐species, using both UCSC Genome Browser and NCBI BLASTN algorithm, indicate that human lnc‐APUE has no homologous transcript in the mouse genome. Although it is impossible to verify the alteration of lnc‐APUE expression and its role in hepatic tumorigenesis using a mouse HCC model, such as DEN‐induced HCC, we have provided some in vivo data from mouse xenograft models and human HCC tissues to validate our findings from cell models. As shown, the growth of mouse xenograft was inhibited by silencing lnc‐APUE in HCC cells, and was promoted by overexpressing wild‐type lnc‐APUE, but was not affected by overexpressing lnc‐APUE with mutation in the miR‐20b‐binding sites. Furthermore, lnc‐APUE upregulation was associated with high levels of E2F1 and its target genes, and was related to HNF4*α* downregulation and correlated with the poor survival of HCC patients. These in vivo data support the conclusion from in vitro cell models that upregulation of lnc‐APUE, resulting from HNF4*α* downregulation, promotes hepatoma growth by upregulating the E2F1 level.

In conclusion, we identify a novel HNF4*α*/lnc‐APUE/miR‐20b/E2F1 regulatory axis and disclose its potential functions, that is, downregulation of HNF4*α* may lead to the upregulation of lnc‐APUE in HCC, whereas lnc‐APUE may work as a cellular sponge to bind miR‐20b and relieve its repression on E2F1 expression, resulting in an increase of E2F1 level and in turn accelerating the G1/S transition and cell proliferation.

## 4 Experimental Section

Additional information is provided in the Supporting Information. All oligonucleotide sequences are listed in Table S2, Supporting Information.

##### Human Tissues

After obtaining adequate informed consent, fresh HCC and the corresponding adjacent noncancerous liver tissues were obtained from patients who undertook tumor resection at Sun Yat‐sen University Cancer Center. All tissues were examined histologically and immediately frozen in liquid nitrogen. No local or systemic therapy was carried out before surgery. After operation, no other anticancer therapy was managed before recurrence. The characteristics of 347 studied subjects are listed in Table S1, Supporting Information. This study was approved by the Institutional Research Ethics Committee at Sun Yat‐sen University Cancer Center.

##### Rapid Amplification of cDNA Ends (RACE)

The 3′‐end and 5′‐end of lnc‐APUE were determined using 3′RACE Kit (Invitrogen, Carlsbad, CA, USA) and 5′‐Full RACE Kit (D315, Takara, Kyoto, Japan), as described previously.^[^ ^42^ ^]^

##### RNA Oligoribonucleotides and Plasmid Construction

Duplexes of small interfering RNA (siRNA), miRNA mimics (miR‐20b, miR‐17), the negative control (NC) RNA for siRNA and miRNA; miR‐20b inhibitor (anti‐miR‐20b), miR‐17 inhibitor (anti‐miR‐17), and the negative control for miRNA inhibitor (anti‐NC) were purchased from RIBOBIO (Guangzhou, China). siRNAs targeting human lnc‐APUE (NR_105 045.1), E2F1 (NM_0 05225), DROSHA (NM_01 3235), DICER1 (NM_0 011 95573), HNF4*α* (NM_178 849), HNF4G (NM_0 04133), and RXRA (NM_0 02957) gene were designated as siAPUE, siE2F1, siDROSHA, siDICER1, siHNF4*α*, siHNF4G, and siRXRA, respectively. The negative control RNA is non‐homologous to any human genome sequence.

Lentivirus expression vectors pCDH‐shNC, pCDH‐shAPUE, pCDH‐shHNF4*α*, pCDH‐APUE, pCDH‐APUE‐mut, pCDH‐APUE‐ORF‐Flag, pCDH‐MPM‐Flag,^[^ ^43^ ^]^ pCDH‐E2F1, pCDH‐HNF4*α*, pCDH‐S1m‐APUE, and pCDH‐S1m‐APUE‐mut were generated on pCDH‐CMV‐MCS‐EF1‐copGFP (System Biosciences, Palo Alto, CA, USA), which expresses copGFP and was denoted as pCDH‐Ctrl.

The firefly luciferase reporter vectors psi‐APUE‐wt, psi‐APUE‐mut, and psi‐E2F1‐3’UTR were constructed based on psiCHECK2 (Promega, Madison, WI, USA), a dual luciferase (*Renilla* and firefly luciferases) expression vector. The vectors P(−1553/+70), P(−889/+70), P(−505/+70), P(−272/+70), P(−212/+70), and P(−140/+70) were constructed based on luciferase reporter vector pGL3‐basic (Promega).

##### Lentivirus Production

To produce lentiviruses, human embryonic kidney cell expressing SV40 large T antigen (HEK293T) cells were co‐transfected with a lentivirus expression vector that carried target sequence and packaging vectors (Lenti‐X HTX Packaging Mix; Clontech, Palo Alto, CA, USA), was then refreshed with a culture medium 16 h post‐transfection, and incubated for an additional 36 h. The lentiviral supernatant was collected and frozen at −80 °C until use.

##### Cell Lines

Human hepatoma cell lines (SK‐Hep‐1, HepG2 and Huh‐7) and HEK293T were grown in Dulbecco's modified Eagle's medium (DMEM; Life Technologies, Carlsbad, CA, USA) containing 10% fetal bovine serum (FBS; Hyclone, Logan, UT, USA).

The stable cell lines were established by infecting human hepatoma cell lines with lentivirus that expressed the target sequence, including sublines stably expressing lnc‐APUE with wild‐type sequence (HepG2‐APUE, Huh‐7‐APUE, SK‐APUE) or with mutant miR‐20b‐binding sites (SK‐APUE‐mut), sublines with stable expression of S1m‐tagged full‐length wild‐type lnc‐APUE (HepG2‐S1m‐APUE), S1m‐tagged full‐length lnc‐APUE with mutant miR‐20b/miR‐17‐binding sites (HepG2‐S1m‐APUE‐mut), E2F1 (Huh‐7‐E2F1) or HNF4*α* (HepG2‐HNF4*α*), and the control lines (HepG2‐Ctrl, Huh‐7‐Ctrl and SK‐Ctrl); and sublines with stable silencing of APUE (SK‐shAPUE) or HNF4*α* (HepG2‐shHNF4*α*‐1 and HepG2‐shHNF4*α*‐2 that expressed different shHNF4*α* sequences) and their control lines SK‐shNC and HepG2‐shNC.

##### Cell Transfection

Ten nm of RNA duplex and 200 nm of miRNA inhibitor were transfected using Lipofectamine RNAiMAX (Invitrogen), and plasmids were transfected using Lipofectamine 3000 (Invitrogen).

##### Analysis of Gene Expression

The levels of genes were determined by real‐time quantitative PCR (qPCR) or western blotting. The intensity for each band in immunoblots was quantified densitometrically. The protein level of a target gene was normalized by the level of *β*‐actin in each sample and the normalized value is shown under each band.

##### Cell Counting Assay

For loss‐of‐function assays, the siRNA‐transfected HepG2 (8 × 10^4^), Huh‐7 (8 × 10^4^), and SK‐Hep‐1 (5 × 10^4^) cells were grown in a 12‐well plate for 96 h before analysis. For gain‐of‐function assays, Huh‐7 subline with stable expression of lnc‐APUE and the control line (Ctrl) (6 × 10^4^) were seeded in a 12‐well plate for 96 h before analysis.

##### Colony Formation Assay

Cells (500 HepG2, 300 Huh‐7 and SK‐Hep‐1 cells) were grown in a 6‐well plate at 37 °C for 2 weeks, followed by fixation in methanol and staining in a 0.1% crystal violet solution for 15 min before colony counting.

##### Cell Cycle Analysis

Cell cycle was analyzed using propidium iodide (PI) staining, followed by fluorescence‐activated cell sorting (FACS) analysis (Gallios, Beckman Coulter, Miami, FL, USA).

##### Ethynyldeoxyuridine (EdU) Assay

The fraction of DNA‐replicating cells, which represents cell proliferation status, was assessed using EdU detection kit (RiboBio, Guangzhou, China). The EdU incorporation rate was calculated as the ratio of the number of EdU‐incorporated cells to the number of Hoechst 33342‐staining cells. At least 500 cells were counted for every group.

##### Luciferase Reporter Assay

Cells were grown in a 48‐well plate with 200 µL complete medium. Luciferase activity was detected 48 h post‐transfection using the dual‐luciferase reporter assay system (Promega). pRL‐TK (Promega) expressing *Renilla* luciferase served as an internal control to correct variances in transfection and harvest efficiency.

To verify the target genes of miRNAs, cells were transfected with 10 nm NC duplex or miRNAs and 25 ng firefly luciferase reporter vectors psi‐APUE‐wt, psi‐APUE‐mut or psi‐E2F1‐3’UTR.

To examine the ceRNA activity of lnc‐APUE, HepG2‐Ctrl or HepG2‐APUE cells were transfected with 10 nm RNA duplex and 25 ng psi‐E2F1‐3’UTR.

To determine the lnc‐APUE promoter region, cells were transfected with 25 ng pRL‐TK and 50 ng P(−1553/+70), P(−889/+70), P(−505/+70), P(−272/+70), P(−212/+70) or P(−140/+70).

To evaluate the role of HNF4*α* expression on the activity of lnc‐APUE promoter, HepG2‐HNF4*α* and its control line HepG2‐Ctrl were transfected with 50 ng P(−212/+70) and 25 ng pRL‐TK. To determine the effect of HNF4*α* knockdown on the activity of lnc‐APUE promoter, HepG2‐shHNF4*α* and its control line HepG2‐shNC were transfected with 50 ng P(−212/+70) and 25 ng pRL‐TK.

##### Mouse Xenograft Models

All mouse experiments were approved by the Institutional Animal Care and Use Committee at Sun Yat‐Sen University. All experiments were conducted according to the Guide for the Care and Use of Laboratory Animals (National Institutes of Health Publication No. 80‐23, revised 1996) and according to the institutional ethical guidelines for animal experiments.

Male NOD‐Prkdcem26Cd52Il2rgem26Cd22/Nju (NCG) mice (4–5 weeks old) were used. For loss‐of‐function study, SK‐shAPUE and its control line SK‐shNC cells (4.0 × 10^6^) were resuspended in 100 µL serum‐free DMEM/Matrigel (1:1), and injected subcutaneously into either side of the armpit. After inoculation for 28 days, the mice were sacrificed and tumors were dissected and weighed. For gain‐of‐function study, SK‐APUE, SK‐APUE‐mut or its control lines SK‐Ctrl cells (4.0 × 10^6^) were suspended in 100 µL serum‐free DMEM/Matrigel (1:1), and injected subcutaneously into the left or right side of the posterior flank. Mice were sacrificed 20 or 22 days after implantation. Tumor volume at different time points was detected with calipers and calculated with formula: volume = length × width^2^/2.

##### Isolation of Cytoplasm and Nuclear Fraction

NE‐PER Nuclear and Cytoplasmic Extraction Reagents (Pierce, Rockford, IL, USA) were used to isolate nuclear/cytoplasmic fractions.

##### RIP Assay

The AGO2‐RNA complex was immunoprecipitated with antibody against AGO2 or isotype‐matched control IgG. The total RNAs were isolated from the precipitates using the TRIzol reagent (Invitrogen) and detected by qPCR.

##### S1m‐Tagged RNA Affinity Purification

miRNA bound to lnc‐APUE was analyzed by affinity purification via S1m‐tag, as described previously.^[^ ^44^ ^]^ S1m‐APUE or S1m‐APUE‐mut was captured by streptavidin Dynabeads (65 001, Invitrogen), and the total RNAs were isolated from the precipitates using the TRIzol reagent (Invitrogen) and the bound miRNAs were detected by qPCR. The untagged APUE was used as a negative control.

##### EMSA and Antibody‐Supershift Assay

EMSA and antibody‐supershift assay were performed using Chemiluminescent EMSA Kit (Beyotime, Shanghai, China). In brief, the biotin‐labeled probes were incubated with nuclear proteins of HepG2 cells for 30 min at room temperature (RT), followed by native‐PAGE. For competition assay, nuclear proteins were pre‐incubated with an unlabeled consensus binding sequence of HNF4*α* before labeled probes were added. For antibody‐supershift assay, nuclear proteins were pre‐incubated with anti‐HNF4*α* antibody (ab181604, Abcam, Cambridge, UK) or isotype‐matched control IgG prior to the addition of labeled probe.

##### ChIP Assay

HepG2 cells were cross‐linked with 0.75% formaldehyde for 15 min and sonicated to shear DNA to ≈200–750 bp. The chromatin‐protein complexes were precipitated with 4 µg antibody against HNF4*α* (Abcam) or isotype‐matched control IgG, then enriched by Protein A/G MagBeads (Bimake, Houston, TX, USA). After extensive washing, the bead‐bound immunocomplexes were eluted using 420 µL elution buffer (0.1 m NaHCO3, 1% SDS). To reverse DNA‐protein crosslinks, 400 µL supernatants were mixed with Tris‐EDTA buffer (16 µL of 1 m Tris‐HCl (pH 6.5), 8 µL of 0.5 M EDTA, 17 µL of 5 m NaCl, 20 µg proteinase K) and heated at 65 °C for 12 h. The immunoprecipitated DNAs fragments were purified and subjected to qPCR.

##### Statistical Analysis

All statistical tests were conducted using GraphPad Prism 5.0 (GraphPad Software, Inc., San Diego, CA, USA). Two tailed unpaired or paired Student′s *t*‐test or two‐way ANOVA were used to compare the differences between two groups. Spearman's correlation coefficient was used to examine the correlation between the levels of different genes in HCC tissues. RFS was calculated from the date of HCC resection to the time of first recurrence or death. Patients lost to follow‐up were treated as censored data. Kaplan–Meier survival curves and Cox proportional hazard regression analyses were performed using SPSS 16.0 (SPSS Inc., Chicago, IL) to identify prognostic factors. Conservation analysis of lnc‐APUE locus across species was performed using UCSC Genome Browser (http://genome.ucsc.edu/) and NCBI blastn algorithm (https://blast.ncbi.nlm.nih.gov/Blast.cgi?PROGRAM=blastn).

The data from at least three independent experiments are presented as mean ± SEM. *p* < 0.05 was defined as significant.

## Conflict of Interest

The authors declare no conflict of interest.

## Author Contributions

S.Y.L. designed and performed experiments, discussed and interpreted the data, wrote the manuscript. R.N.L., J.H.H., and K.Y. performed experiments and interpreted the data. Y.F.Y., S.M.Z., and Y.Z. supervised and designed the study, discussed and interpreted the data, wrote the manuscript. All authors read and approved the final manuscript.

## Supporting information

Supporting Information
