# Supplementary material for: LncRNA Lnc‐APUE is Repressed by HNF4α and Promotes G1/S Phase Transition and Tumor Growth by Regulating MiR‐20b/E2F1 Axis
Source: Adv Sci (Weinh). 2021 Feb 1;8(7):2003094. doi: 10.1002/advs.202003094 (PMC8025008; doi:10.1002/advs.202003094)
Supplement: Supplementary file 1 — Supporting Information [file ADVS-8-2003094-s001.pdf]

© 2021 Wiley-VCH GmbH

ADVANCED SCIENCE
Open Access

Supporting Information

for *Adv. Sci.*, DOI: 10.1002/advs.202003094

LncRNA Lnc-APUE Is Repressed by HNF4α and Promotes G1/S Phase Transition and Tumor Growth by Regulating MiR-20b/E2F1 Axis

Song-Yang Li, Ying Zhu, Ruo-Nan Li, Jia-Hui Huang, Kai You, Yun-Fei Yuan, Shi-Mei Zhuang**

Supporting Information

LncRNA Lnc-APUE Is Repressed by HNF4α and Promotes G1/S Phase Transition and Tumor Growth by Regulating MiR-20b/E2F1 Axis

Song-Yang Li, Ying Zhu, Ruo-Nan Li, Jia-Hui Huang, Kai You, Yun-Fei Yuan, Shi-Mei Zhuang**

Inventory of supporting data

1. Supplementary Experimental Section...……………..Page 3 - 9

2. Supplementary figures and legends…………………Page 10 - 21

3. Supplementary tables………………….……..……… Page 22 - 28

Supplementary Experimental Section

Rapid amplification of cDNA ends (RACE): The 3'-end of lnc-APUE transcript was confirmed using a 3'RACE System (Version 2.0; Invitrogen, Carlsbad, CA, USA). Total RNAs from noncancerous liver tissues were subjected to reverse transcription with a 3'RACE adaptor primer. The tailed cDNA was then amplified using gene specific primer 1 (GSP1) and a 3'RACE inner primer, followed by a second PCR with GSP2 and a 3'RACE inner primer.

The 5'-end of lnc-APUE transcript was determined using a 5'-Full RACE Kit (D315, TaKaRa, Kyoto, Japan). Total RNAs from noncancerous liver tissues were treated with calf intestinal phosphatase (CIAP) and tobacco acid pyrophosphatase (TAP), and then ligated to a 5'RACE RNA adaptor, followed by reverse-transcription with random primers. The cDNA was then amplified using GSP3 and a 5'RACE outer primer, followed by a second PCR with GSP4 and a 5'RACE inner primer.

The PCR product was verified by direct sequencing.

Plasmid construction: The following expression vectors were used: lentivirus expression vectors, including pCDH-APUE, pCDH-APUE-mut, pCDH-S1m-APUE, pCDH-S1m-APUE-mut, pCDH-E2F1, pCDH-HNF4α, pCDH-shNC, pCDH-shAPUE, pCDH-shHNF4α-1 and pCDH-shHNF4α-2; firefly luciferase reporters, including psi-APUE-wt, psi-APUE-mut, psi-E2F1-3'UTR, P(−1553/+70), P(−889/+70), P(−505/+70), P(−272/+70), P(−212/+70), P(−140/+70).

To create pCDH-APUE or pCDH-APUE-mut, full-length lnc-APUE (1123-nt) with

wild-type sequence or with mutant miR-20b-binding sites was inserted into the *EcoR*I/*Swa*I sites of the lentivirus expression vector pCDH-CMV-MCS-EF1-copGFP (System Biosciences, Palo Alto, CA, USA), which contained a copGFP expression cassette and was designated pCDH-Ctrl in this study. To create pCDH-E2F1 and pCDH-HNF4α, the coding sequence of E2F1 and HNF4α were respectively inserted into the *EcoR*I/*BamH*I sites of pCDH-Ctrl.

To perform S1m-tagged RNA affinity purification, pCDH-S1m was constructed by inserting four tandem S1m sequences, which encoded the streptavidin-binding RNA aptamer, into the *Xba*I/*EcoR*I sites in pCDH-Ctrl. Full-length lnc-APUE with wild-type sequence or with mutant miR-20b-binding sites was then inserted into the *EcoR*I/*Swa*I sites downstream of the 4×S1m sequence in pCDH-S1m to generate pCDH-S1m-APUE or pCDH-S1m-APUE-mut.

To construct pCDH-shNC and pCDH-shAPUE vectors, the complementary oligonucleotides that contained both sense and antisense siRNA sequences, the spacer sequence (5’-CTCGAG) and the flanking *EcoR*I and *BamH*I sites were chemically synthesized, annealed and then inserted into the *EcoR*I/*BamH*I sites in the pCDH-U6 vector, which was produced based on the pCDH-CMV-MCS-EF1-copGFP vector by replacing the CMV promoter with the U6 promoter.

To verify whether miR-20b and miR-17 targeted lnc-APUE, psi-APUE-wt and psi-APUE-mut were constructed. Full-length lnc-APUE with wild-type sequence or mutant miR-20b-binding sites was inserted into the *Xho*I/*Not*I sites downstream of the

stop codon of *Renilla* luciferase in psiCHECK2 (Promega, Madison, WI, USA), which expressed firefly luciferase as an internal control. To verify whether miR-20b targeted the 3′untranslated region (3'UTR) of E2F1, a 3'UTR segment of E2F1 mRNA that contained the putative miR-20b-binding sites was inserted into the *Xho*I/*Not*I sites downstream of the stop codon of *Renilla* luciferase in psiCHECK2 to generate psi-E2F1-3'UTR.

To construct the firefly luciferase reporter plasmids for verifying the lnc-APUE promoter region, the genomic fragments upstream of lnc-APUE were cloned into the *Kpn*I/*Hind*III sites upstream of the firefly luciferase gene in pGL3-basic vector (Promega).

All constructs were verified by sequencing. The sequences of primers are listed in Table S2, Supporting Information.

Analysis of gene expression: Real-time quantitative polymerase chain reaction (qPCR) was used to evaluate the RNA levels. Total RNAs were isolated by TRIzol Kit (Life Technologies, Carlsbad, CA, USA), and the RNA quantity was measured using a NanoDrop equipment (Thermo Fisher Scientific, Waltham, MA, USA). For mRNA and lncRNA analysis, 2 μg of total RNA was subjected to DNase I digestion (Fermentas, Hanover MD, USA) at 37 °C for 30 minutes and then to heat inactivation at 65 °C for 10 minutes, followed by reverse-transcription using M-MLV (M1701, Promega). qPCR was performed on a LightCycler 480 (Roche Diagnostics, Germany) using 2×SYBR Green qPCR Master Mix (B21202, Bimake, Houston, TX, USA). All

reactions were performed in duplicate. The cycle threshold (Ct) values differed by less than 0.5 between duplicates. All target genes were normalized to that of endogenous reference gene, which yielded a $2^{-\Delta Ct}$ value. U6 and β-actin were used as the reference genes for the relative expression levels in tissues and cell lines, respectively.

For qPCR analysis of miRNA, the levels of miR-20b, miR-17 and miR-378g were detected using a Bulge-Loop™ miRNA qRT-PCR Starter Kit (Ribobio, Guangzhou, China).

Western blotting was conducted to determine the protein levels. Samples were separated on 8 ~ 12% SDS PAGE, transferred to polyvinylidene difluoride membranes (162-0177, Bio-Rad, Hercules, CA, USA). and then detected with antibodies. The following antibodies were used: rabbit monoclonal antibodies (mAbs) against human phospho-Ser807/811 of pRb (9308), β-actin (4970), E2F1 (3742), cyclin E2 (4132), pRb (9309), CDK4 (12790), CDK6 (13331), cyclin D1 (2922), CDK2 (2546), p15 (4822), p16 (80772) from Cell Signalling Technology (CST, Beverly, MA, USA); rabbit mAbs against cyclin E1 (ENT1176, Elabscience, Wuhan, China). The intensity of the specific band for target protein was detected using Image J software (National Institute of Health, Bethesda, MD, USA).

Cell cycle analysis: Cell cycle distribution was profiled using propidium iodide (PI) staining followed by fluorescence-activated cell sorting (FACS) analysis. For nocodazole-synchronized model, cells were transfected with the indicated siRNA duplex for 36 hours and then treated with 40 ng/mL nocodazole for 12 hours. For serum starvation-stimulation assays, cells transfected with the indicated siRNAs were serum-deprived for 48 hours, followed by serum re-addition and then incubation for the indicated times. The cells were then collected and resuspended in a

detergent-containing hypotonic solution (Krishan's reagent: 0.05 mg/mL propidium iodide, 0.1% Na-citrate, 0.02 mg/mL ribonuclease A, 0.3% NP-40), followed by incubation at 37 °C for 30 minutes and subsequent FACS analysis using cytometer (Gallios, Beckman Coulter, Miami, FL, USA).

RNA immunoprecipitation (RIP) assay: HepG2 cells (1×10^7) were washed with 5 mL 1× PBS three times, cross-linked with 0.5% formaldehyde at room temperature (RT) for 10 minutes to crosslink cellular components; the reaction was aborted in 250 mM glycine at RT for 5 minutes. Cells were washed with 5 mL ice-cold 1×PBS three times, dissolved with RNase free RIP lysis buffer (25 mM Tris-HCl at pH 7.4, 150 mM NaCl, 1 mM EDTA, 1% NP-40, 5% glycerol, protease inhibitor cocktail) and incubated at 4 °C for 10 minutes. The samples were sonicated for 4 times with a Bioruptor (Diogenode, Liege, Belgium), followed by centrifugation at 13,000×g, 4 °C for 10 minutes. The supernatants were pre-cleared by 20 μL of Dynabeads Protein G (10004D, Invitrogen), then incubated with 4 μg of antibody at 4 °C for 4 hours, followed by addition of 40 μL Dynabeads G (Invitrogen) and further incubation for 2 hours. The beads were washed with a high-salt RIP lysis buffer (25 mM Tris-HCl at pH 7.4, 500 mM NaCl, 1 mM EDTA, 1% NP-40 and 5% glycerol) five times and then dissolved with 50 μL of 100 mM glycine (pH 3.0) to release the immunoprecipitated complex. Then, 5 μL of 1 M Tris-HCl (pH 8.0) and 5 μg proteinase K were added to the immunoprecipitates and incubated sequentially at 55 ℃ for 1 hour and at 70 °C for 45 minutes to reverse the formaldehyde crosslinking. Total RNAs were extracted from the eluent, followed by qPCR analysis for target genes.

The antibodies used for the RIP assay included mouse mAb against AGO2 (H00027161-M01, Abnova, Taipei, Taiwan) and isotype-matched mouse control IgG (A7028, Beyotime, Shanghai, China).

Purification of S1m-tagged RNA-RNA complexes: HepG2 (2×10^7) sublines with stable expression of APUE, S1m-APUE or S1m-APUE-mut were washed with 5 mL 1×PBS three times, pelleted by centrifugation at 500×g for 5 minutes, resuspended in 8 mL 1×PBS containing 0.37% formaldehyde and rotated at RT for 5 minutes to crosslink cellular components, followed by incubation with 250 mM glycine at RT for another 5 minutes to abort crosslinking reaction. Cell pellets were washed 2 times with 5 mL ice-cold 1×PBS, dissolved with ice-cold RNase free lysis buffer (150 mM NaCl, 10 mM HEPES at pH 7.4, 3 mM MgCl2, 10% glycerol, 1% NP-40, 2 mM DTT, 1 mM PMSF and protease inhibitor cocktail), followed by incubation on ice for 10 minutes, and then sonicated six times with a Bioruptor. The supernatant was collected by centrifugation (18,000×g, 4 °C, 10 minutes) and pre-cleared by incubation with 40 μL avidin agarose beads (20219, Pierce, Rockford, IL, USA) at 4 °C for 1 hour; then, 30 μL streptavidin Dynabeads (Invitrogen) was added, and the sample was rotated at 4 °C for 3 hours to pulldown S1m-tagged RNA-RNA complexes. The beads were washed five times with 500 μL ice-cold RNase-free washing buffer (50 mM HEPES at pH 7.4, 400 mM NaCl, 0.1% TritonX-100, 10% glycerol, 2% NP-40, 1 mM EDTA, 1 mM PMSF, and 1 mM DTT) by rotation for 5 minutes each at 4 °C, followed by adding 100 μL RNase-free elution buffer (50 mM HEPES at pH 7.4, 5 mM EDTA, 100 mM NaCl, 1% SDS and 10 mM DTT) and heating at 70 °C for 45 minutes to

reverse formaldehyde crosslinking. RNAs were extracted from the precipitates by TRIzol reagent (Invitrogen) and detected by qPCR.

Supplementary figures and legends

Figure S1

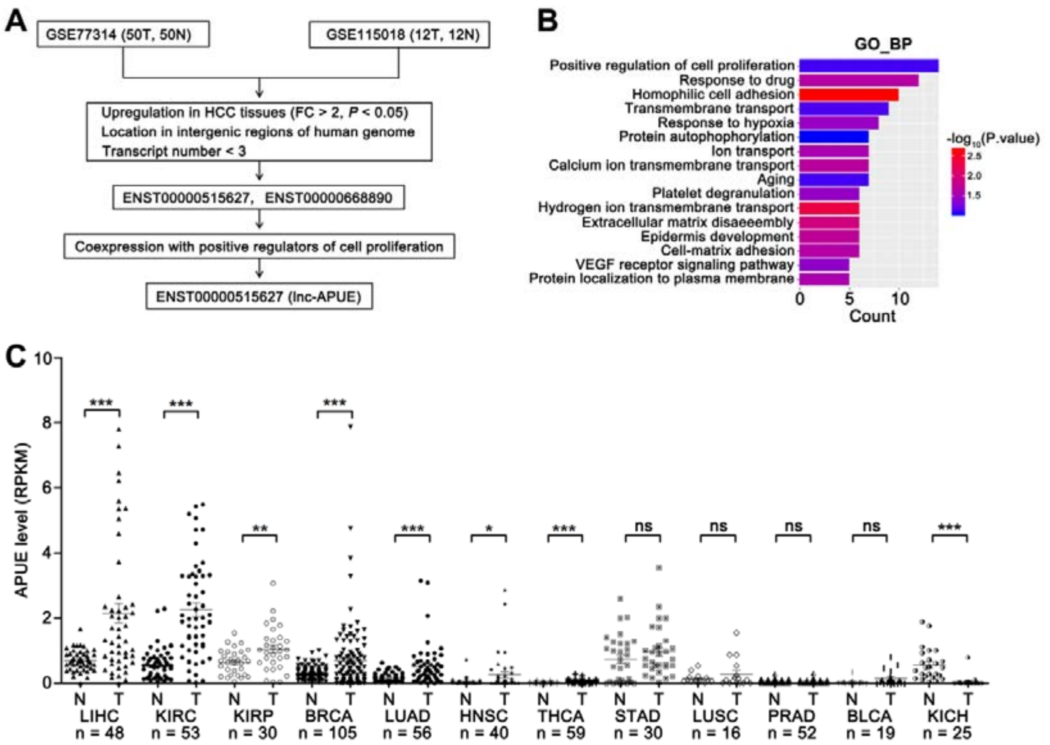

Figure S1. The screening for oncogenic lncRNAs. A) The screening workflow for candidate lncRNAs. LncRNA expression profilings of paired HCC and noncancerous liver tissues were derived from National Center for Biotechnology Information (NCBI) Gene Expression Omnibus (GEO, accession number GSE77314 and GSE115018). B) Gene Ontology (GO) analysis for the protein-coding genes co-expressed with lnc-APUE. The genes that were co-expressed with lnc-APUE were obtained from TANRIC website (https://ibl.mdanderson.org/tanric/_design/basic/ query.html). GO analysis was performed in GO_BP of DAVID (https://david.ncifcrf.gov/). C) Pan-cancer analysis of lnc-APUE expression based on TCGA data. The expression levels of lnc-APUE in paired tumor and nontumor tissues from 12 different cancer types are presented. RPKM, reads per kilobase per million mapped reads. N,

noncancerous tissue; T, tumor tissue. LIHC, liver hepatocellular carcinoma; KIRC, kidney renal clear cell carcinoma; KIRP, kidney renal papillary cell carcinoma; BRCA, breast invasive carcinoma; LUAD, lung adenocarcinoma; HNSC, head and neck squamous cell carcinoma; THCA, thyroid carcinoma; STAD, stomach adenocarcinoma; LUSC, lung squamous cell carcinoma; PRAD, prostate adenocarcinoma; BLCA, bladder urothelial carcinoma; KICH, kidney chromophobe. n, the number of paired tissues. For (C), the data are presented as mean ± SEM; P values were assessed by paired Student′s t-test *, $P < 0.05$; **, $P < 0.01$; ***, $P < 0.001$; ns, not significant.

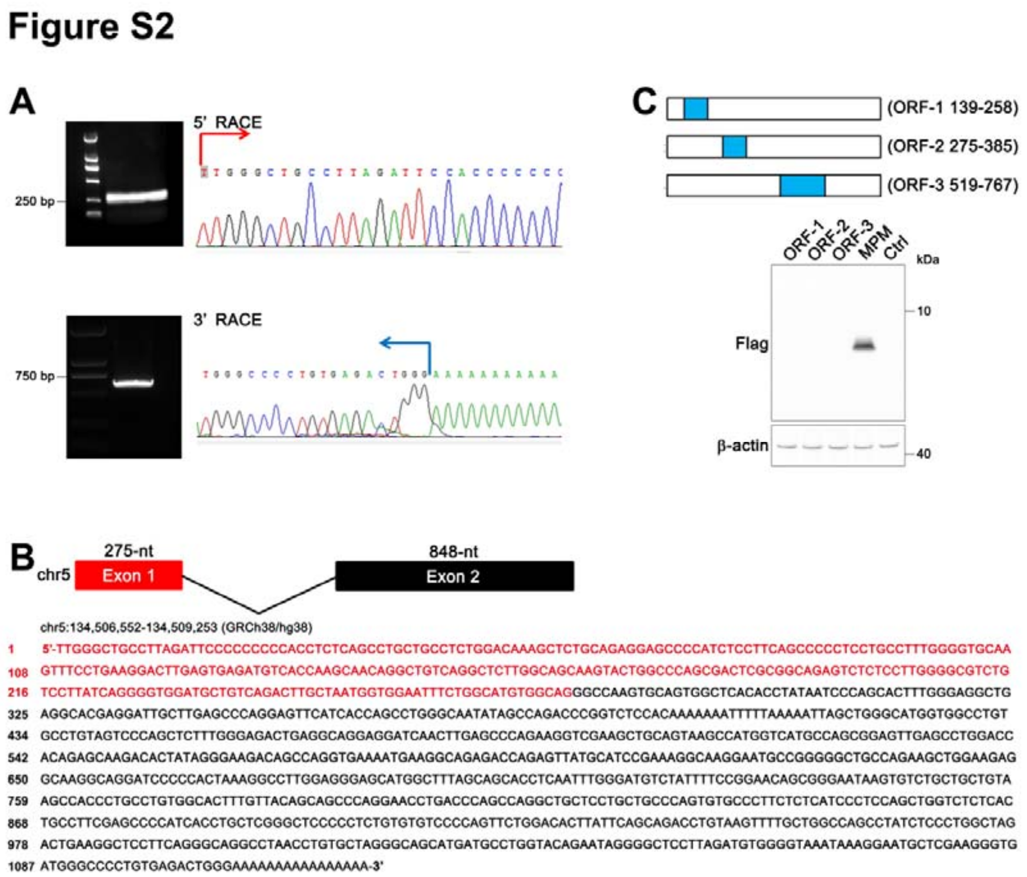

Figure S2. Characterization of the lnc-APUE transcript. A) The PCR products of RACE. The products of 5'RACE and 3'RACE were obtained by nested-PCR and then sequenced. The sequences of the 5'- or 3'-ends of lnc-APUE transcript are marked. B) Schematic diagram and nucleotide sequences of lnc-APUE. The lnc-APUE transcript consists of two exons (the first exon in red font and the second in black font). C) The lnc-APUE transcript had no protein-coding capacity. The putative open reading frames of lnc-APUE were predicted by NCBI ORFfinder (https://www.ncbi.nlm.nih.gov/orffinder) and fused with Flag (without ATG) in pCDH-CMV-MCS-EF1-copGFP (pCDH-Ctrl), then transfected into 293T cells, followed by immunoblotting using anti-Flag antibody. MPM is a

mitochondria-localized micropeptide and the MPM-Flag fusion plasmid was used as a positive control and the pCDH-Ctrl-Flag fusion plasmid as a negative control.

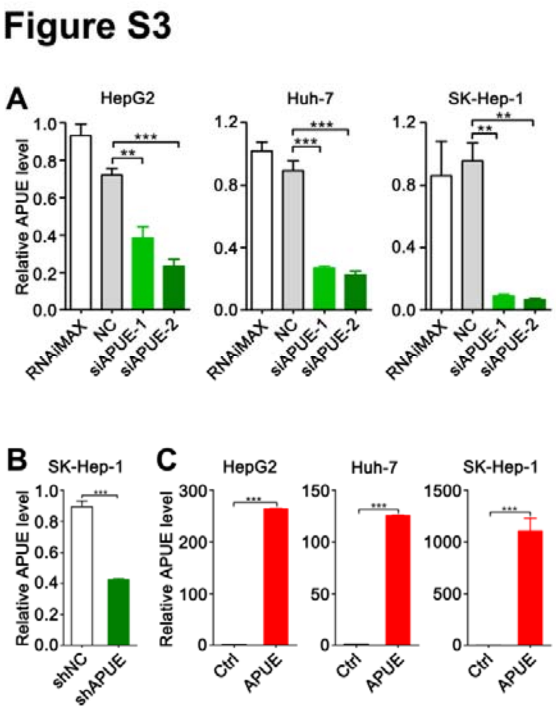

Figure S3. The knockdown and overexpression efficiency of lnc-APUE. A) The level of cellular lnc-APUE was reduced by siAPUE. HepG2, Huh-7 and SK-Hep-1 cells were transfected with NC, siAPUE-1 or siAPUE-2 for 36 hours before qPCR analysis. B) The level of cellular lnc-APUE was reduced by shRNA targeting lnc-APUE. SK-Hep-1 cells were infected with lentiviruses carrying pCDH-shAPUE or pCDH-shNC for 96 hours before qPCR analysis. C) Ectopic expression of lnc-APUE. HepG2, Huh-7 and SK-Hep-1 cells were infected with lentiviruses carrying pCDH-APUE or pCDH-Ctrl for 96 hours before qPCR analysis. For (A-C), the data from at least three independent experiments are presented as mean ± SEM; *P* values were assessed by unpaired Student′s *t*-test. **, $P < 0.01$; ***, $P < 0.001$.

Figure S4

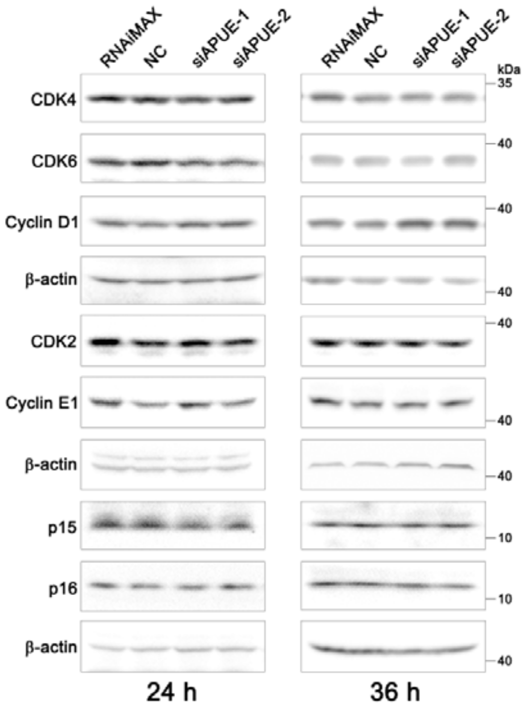

Figure S4. The impact of siAPUE on the levels of key regulators of pRb phosphorylation. HepG2 cells were exposed to RNAiMAX or transfected with NC or siAPUE for 24 hours or 36 hours before immunoblotting.

Figure S5

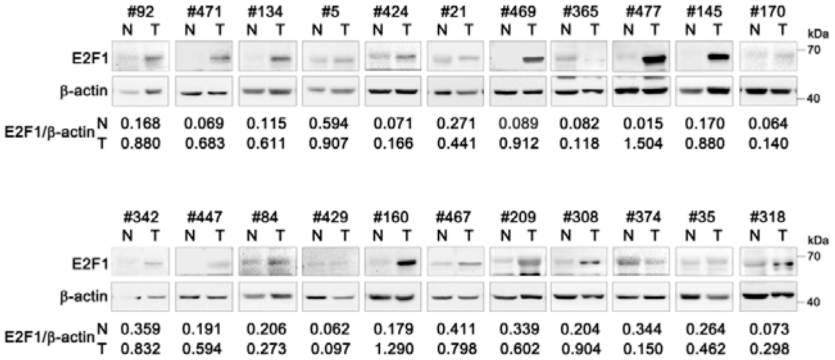

Figure S5. Detection of E2F1 in human HCC tissues. The levels of E2F1 were assessed by immunoblotting in 22 paired HCC (T) and adjacent noncancerous liver tissues (N). The level of E2F1 relative to β-actin level is indicated under each band.

Figure S6

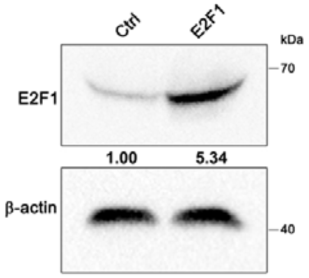

Figure S6. Ectopic expression of E2F1 in Huh-7 cells. Cells were infected with lentiviruses carrying pCDH-Ctrl or pCDH-E2F1, then subjected to immunoblotting at 96 hours post-infection. The level of E2F1 relative to β-actin level is indicated under each band.

Figure S7

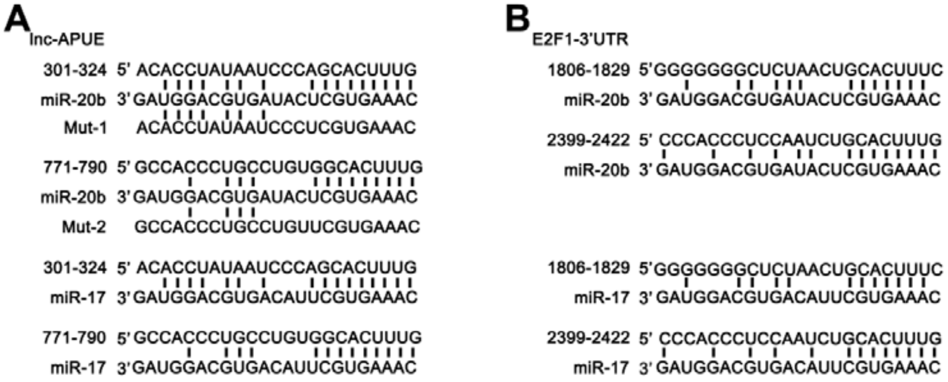

Figure S7. The putative binding sites of miR-20b and miR-17 in lnc-APUE and E2F1-3'UTR. Short vertical lines indicate the paired bases between miRNAs and their target sites in A) lnc-APUE and B) E2F1-3'UTR. Mutations were generated in the complementary site that binds to the seed region of miR-20b.

Figure S8

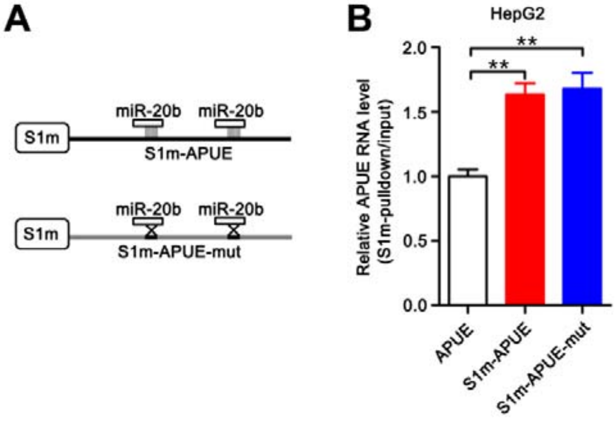

Figure S8. Lnc-APUE was enriched in the precipitates from S1m-APUE- and S1m-APUE-mut-transfected cells. A) The schematic diagram of S1m-APUE and S1m-APUE-mut construct. The short vertical lines indicate the binding sites of miR-20b in S1m-APUE, while the crosses depict the mutated sites in S1m-APUE-mut. B) HepG2-APUE, HepG2-S1m-APUE and HepG2-S1m-APUE-mut sublines were subjected to S1m-tagged RNA affinity purification. Lnc-APUE in the S1m-pulldown product and the input were analysed by qPCR. The levels of lnc-APUE in the pulldown product were adjusted by that in the input. The mean value of the adjusted RNA level in the pulldown product of the HepG2-APUE group from three experiments was set as relative RNA level 1. For (B), the data from at least three independent experiments are presented as mean ± SEM; *P* values were assessed by unpaired Student's *t*-test. **, $P < 0.01$.

Figure S9

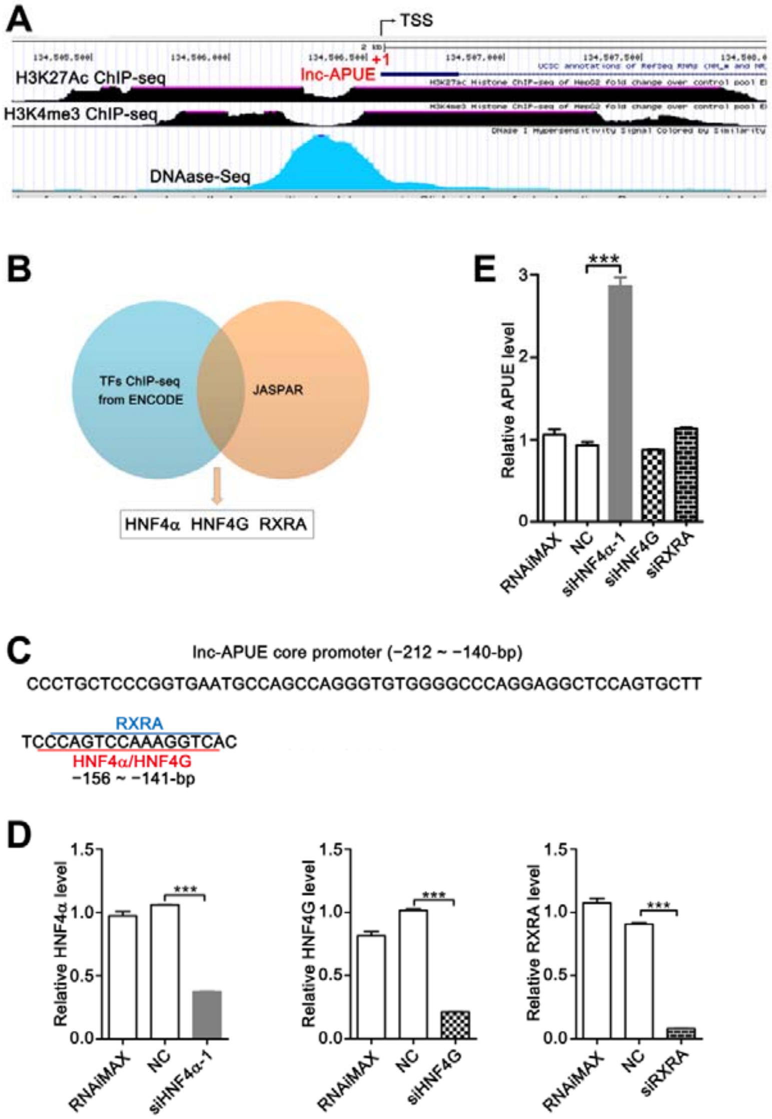

Figure S9. Predicition of transcription factors that may regulate lnc-APUE expression. A) The profiles of H3K4me3, H3K27Ac and DNase I hypersensitive signal were enriched within the 1500-bp genomic region upstream of the transcriptional start site (TSS, assigned as +1) of lnc-APUE. Arrow indicates the direction of transcription. B) Transcription factors that may regulate lnc-APUE expression were predicted by ChIP-seq from ENCODE (https://www.encodeproject.org/) and JASPAR

(http://jaspar.binf.ku.dk/) website. C) Putative HNF4α, HNF4G and RXRA binding sites in the core promoter of lnc-APUE. D) Silencing HNF4α, HNF4G and RXRA in HepG2 cells. RNAiMAX, cells exposed to Lipofectamine RNAiMAX but not RNA duplexes. NC, cells transfected with the negative control RNA duplex. siHNF4α, siHNF4G and siRXRA, cells transfected with siRNA targeting HNF4α, HNF4G and RXRA, respectively. E) HNF4α knockdown elevated the level of lnc-APUE. HepG2 cells were transfected with the indicated siRNAs for 48 hours before qPCR analysis. For (D-E), the data from at least three independent experiments are presented as mean ± SEM; *P* values were assessed by unpaired Student′s *t*-test. ***, $P < 0.001$.

Figure S10

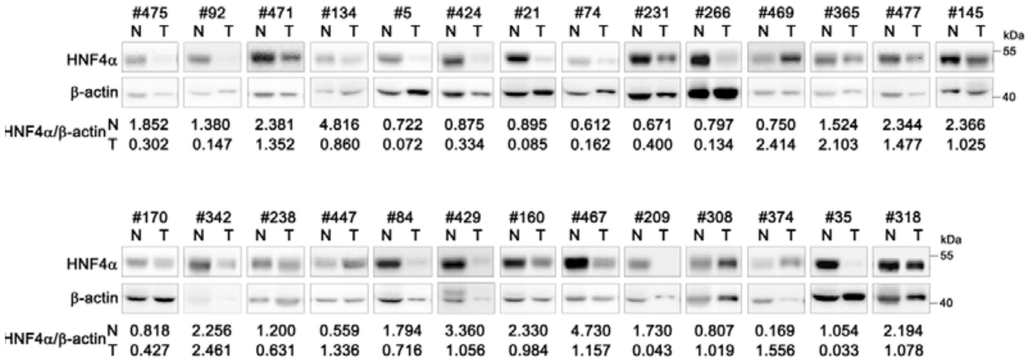

Figure S10. Downregulation of HNF4α in human HCC tissues. The protein levels of HNF4α were assessed by immunoblotting in 27 paired HCC (T) and adjacent noncancerous liver tissues (N). The level of HNF4α relative to β-actin level is indicated under each band.

Supplementary tables

Table S1. Univariate and multivariate analysis of factors associated with recurrence-free survival

Clinical Variables	Case Number	HR (95% CI)[a]	p^a
**Univariate analysis**			
**lnc-APUE (High vs Low)[b]**	110/237	1.367 (1.012-1.847)	**0.042**
Age (>50 years vs ≤50 years)	183/164	0.767 (0.573-1.026)	0.074
Gender (male vs female)	293/54	1.060 (0.704-1.596)	0.779
HBV infection (+ vs -)[c]	293/54	1.150 (0.764-1.731)	0.504
Tumor size (>5 cm vs ≤5 cm)	182/163	1.513 (1.126-2.032)	**0.006**
Tumor number (Multiple vs Single)	65/280	1.959 (1.405-2.731)	**<0.001**
Tumor capsule (- vs +)	195/151	1.397 (1.039-1.879)	**0.027**
Cirrhosis (+ vs -)	284/61	1.420 (0.925-2.182)	0.109
Alpha-fetoprotein (>20 ng/mL vs ≤20 ng/mL)	217/129	1.388 (1.018-1.892)	**0.038**
Portal vein tumor thrombosis (+ vs -)	22/323	2.028 (1.194-3.444)	**0.009**
Edmondson grade (III / IV vs I / II)	126/219	1.553 (1.155-2.088)	**0.004**
BCLC classification (B/C vs 0/A)	87/260	2.051 (1.504-2.796)	**<0.001**
**Multivariate analysis**			
**lnc-APUE (High vs Low)**	110/237	1.394 (1.027-1.892)	**0.033**
Edmondson grade (III / IV vs I / II)	126/219	1.437 (1.066-1.939)	**0.018**
BCLC classification (B/C vs 0/A)	87/260	2.076 (1.518-2.838)	**<0.001**

[a] HR (hazard ratio) and *p* values were calculated using univariate or multivariate Cox proportional hazards regression; 95% CI, 95% confidence interval.

[b] lnc-APUE level was examined in 347 HCC tissues by qPCR and normalized to U6 level. The 32th percentile value of the examined samples was chosen as the cut-off point to separate lnc-APUE-low from lnc-APUE-high expression groups.

[c] +, presence; -, absence.

Table S2. Sequences of RNA and DNA oligonucleotides

Name	Sense strand/Sense primer (5'-3')	Antisense strand/Antisense primer (5'-3')
**Primers for qPCR**		
lnc-APUE (226-388)[a]	GGGTGGATGCTGTCAGACTT	ACCGGGTCTGGCTATATTGC
CCNA2 (1316-1539)	TTATTGCTGGAGCTGCCTTT	CTCTGGTGGGTTGAGGAGAG
CCNE2 (329-563)	CAGATAATCCAGGCCAAGAA	CAGGCAAAGGTGAAGGATTA
CCNB1 (1061-1276)	GATTGGAGAGGTTGATGTCG	AGGTGCTGCATAACTGGAAG
MYBL2 (626-898)	ATCATCTGCGAGGCCCAC	TATGGTAGGAGGGACGGAGG
MCM3 (476-675)	AGTTCGTCCCAAAGTCGTCC	CCTGGATGGTGATGGTCTGG
CDC6 (291-477)	AAGCTGTCTCGGGCATTGAA	TGCCTTGCTTTGGTGGAGAA
TK1 (374-532)	TGCTCAGTACAAGTGCCTGG	TCGTCGATGCCTATGACAGC
HNF4α (73-291)	ATGCGACTCTCCAAAACCCT	ACCGTAGTGTTTGCCCGT
APOC3 (175-294)	AAGACCGCCAAGGATGCAC	CTTGTCCTTAACGGTGCTCCA
HNF4G (910-1076)	CGTGTGGCCAATCGTGTTC	CCGATCTGCACTTGGAACCT
RXRA (1889-2068)	CTGACGGAGCTTGTGTCCAA	TGGGTACTTGTGCTTGCAGT
MALAT1 (2988-3202)	GAAAGCGAGTGGTTGGTAAA	CCCTCAAAAGCTTCAGACAA
U6 (4-97)	CTCGCTTCGGCAGCACA	AACGCTTCACGAATTTGCGA
β-actin (301-676)	AAGATGACCCAGATCATGTTTGAG	GCAGCTCGTAGCTCTTCTCCAG

Table S2. Sequences of RNA and DNA oligonucleotides (Continued)

Name	Sense strand/Sense primer (5'-3')	Antisense strand/Antisense primer (5'-3')
**siRNA duplexes**		
NC	UUCUCCGAACGUGUCACGUdTdT	ACGUGACACGUUCGGAGAAdTdT
siAPUE-1	GCAAGACACUAUAGGGAAGdTdT	CUUCCCUAUAGUGUCUUGCdTdC
siAPUE-2	CAUCCGAAAGGCAAGGAAUdTdT	AUUCCUUGCCUUUCGGAUGdCdA
siE2F1-1	CACUGAAUCUGACCACCAAdTdT	UUGGUGGUCAGAUUCAGUGdAdG
siE2F1-2	GUGAUUUAUUUAUUGGGAAdTdT	UUCCCAAUAAAUAAAUCACdCdC
siDROSHA	AACGAGUAGGCUUCGUGACUUdTdT	AAGUCACGAAGCCUACUCGUUdCdC
siDICER1	GGACCAUUUACUGACAGAAdTdT	UUCUGUCAGUAAAUGGUCCdAdC
siHNF4α-1	GAUUGCCAGCAUCGCAGAUdTdT	AUCUGCGAUGCUGGCAAUCdTdT
siHNF4G	GCACUGACAUAAACGUUAAdTdT	UUAACGUUUAUGUCAGUGCdTdT
siRXRA	AGACCUACGUGGAGGCAAAdTdT	UUUGCCUCCACGUAGGUCUdCdG
**miRNA mimics**		
miR-20b	CAAAGUGCUCAUAGUGCAGGUAG	ACCUGCACUAUGAGCCACUUUGUU
miR-17	CAAAGUGCUUACAGUGCAGGUAG	ACCUGCACUGUAAGCACUUUGUU

Table S2. Sequences of RNA and DNA oligonucleotides (Continued)

Name	Sense strand/Sense primer (5'-3')	Antisense strand/Antisense primer (5'-3')
**miRNA inhibitors**		
anti-NC	GUGGAUAUUGUUGCCAUCA	
anti-miR-20b	CUACCUGCACUAUGAGCACUUUG	
anti-miR-17	CUACCUGCACUGUAAGCACUUUG	
**Primers for RACE**		
**3'RACE**		
3'RACE-adaptor		GGCCACGCGTCGACTAGTACTTTTTTTTTTTTTTTTT
3'RACE inner primer		GGCCACGCGTCGACTAGTA
GSP1	CAGGGGTGGATGCTGTCAGACTTG	
GSP2	CCATGGTCATGCCAGCGGAGTTGA	
**5'RACE**		
5'RACE inner primer	CGCGGATCCACAGCCTACTGATGATCAGTCGATG	
5'RACE outer prime	CATGGCTACATGCTGACAGCCTA	
GSP3		CAGCAGCAGACACTTATTCCCGCT
GSP4		GAGCCTTCAGTCTAGCCAGGGAGA

Table S2. Sequences of RNA and DNA oligonucleotides (Continued)

Name	Sense strand/Sense primer (5'-3')	Antisense strand/Antisense primer (5'-3')
**Cloning into pCDH-CMV-MCS-EF1-copGFP**		
lnc-APUE	GCTCTAGAGCTGGGGTCATTGGGCTGCCTTAGATTCCCCCCCCCACCTCTCA	GGAATTCCCCCAGTCTCACAGGGGCCCATCACCCTTCGAGC ATTCCT
lnc-APUE-mut-1	GGACGCTGTTCGTGAAACTTACAGCAGCCCAGGAACCT	GTTTCACGAACAGCGTCCGTGGCTTACAGCAGCAGA
lnc-APUE-mut-2	ACGGAGGCTGAGGCACGAGGATTGCT	TCAGCCTCCGTTTCACGTGGGATT
lnc-APUE-ORF1	GCTCTAGAGCTGGGGTCATTGGGCTGCCTT	GGAATTCCTTACTTATCGTCGTCATCCTTGTAATCGCAAGTC TGACAGCATCCA
lnc-APUE-ORF2	GCTCTAGAGCTGGGGTCATTGGGCTGCCTT	GGAATTCCCTACTTATCGTCGTCATCCTTGTAATCTATTGCC CAGGCTGGTGATG
lnc-APUE-ORF3	GCTCTAGAGCTGGGGTCATTGGGCTGCCTT	GGAATTCCTTACTTATCGTCGTCATCCTTGTAATCCAGCAGC AGACACTTATTCC
shAPUE	CATCCGAAAGGCAAGGAAT	ATTCCTTGCCTTTCGGATG
shHNF4α-1	GATTGCCAGCATCGCAGAT	ATCTGCGATGCTGGCAATC
shHNF4α-2	GCTGCAGATCGATGACAATGA	TCATTGTCATCGATCTGCAGC
E2F1	AGTGAATTCGCCACCATGGCCTTGGCCGGGGCC	AGTGGATCCTCAGAAATCCAGGGGGGTGAGGTC
MPM	CGAGAATTCATGGCGGACGTGTCTGAGAG	ACGGGATCCTCACTTGTCATCGTCATCCTTGTAATCGGCCA GGTCCAGCTTTT
HNF4α	GGAATTCCGCCACCATGCGACTCTCCAAAACCCT	CGGGATCCCGTCACTACTTATCGTCGTCATCCTTGTAATCG ATAACTTCCTGCTT
**Cloning into pGL3-basic**		
P(-1553/+70)	GGGGTACCCCGCATCAGGTTCAAAGCCAGG	CCCAAGCTTGGGTGCAGAGCTTTGTCCAGAGG

Table S2. Sequences of RNA and DNA oligonucleotides (Continued)

Name	Sense strand/Sense primer (5'-3')	Antisense strand/Antisense primer (5'-3')
P(-889/+70)	GGGGTACCCCAGATCGCACCATGGCACT	CCCAAGCTTGGGTGCAGAGCTTTGTCCAGAGG
P(-505/+70)	GGGGTACCCCGTCTTGTTGGGACTCTCGGG	CCCAAGCTTGGGTGCAGAGCTTTGTCCAGAGG
P(-272/+70)	GGGGTACCCCACTGGCTACTTGAGGACACC	CCCAAGCTTGGGTGCAGAGCTTTGTCCAGAGG
P(-212/+70)	GGGGTACCCCCCCTGCTCCCGGTGAAT	CCCAAGCTTGGGTGCAGAGCTTTGTCCAGAGG
P(-140/+70)	GGGGTACCCCCTAACTGCCCAGCCGCTG	CCCAAGCTTGGGTGCAGAGCTTTGTCCAGAGG
**Primers for ChIP**		
lnc-APUE	TCCCTGCTCCCGGTGAAT	CCCTGCACACACTCCCAG
APOC3	CCAGGAAGGTGAACGAGAGA	GCTCCCTATTTTGCCCTCTG
GAPDH	TACTAGCGGTTTTACGGGCGCACGT	AACAGGAGGAGCAGAGAGCGAA
**Probes for EMSA**		
lnc-APUE	TTTCCCAGTCCAAAGGTCACTAA	TTAGTGACCTTTGGACTGGGAAA
HNF4α-consensus	AGTGAGGGTCAAAGTTCATATCAC	GTGATATGAACTTTGACCCTCACT
non-specific oligo	AGTGAGAGTTGGCTCGAGGCGCAC	GTGCGCCTCGAGCCAACTCTCACT

Table S2. Sequences of RNA and DNA oligonucleotides (Continued)

Name	Sense strand/Sense primer (5'-3')	Antisense strand/Antisense primer (5'-3')
**Cloning into psiCHECK2**		
lnc-APUE	AGTCTCGAGTGGGGTCATTGGGCTGCCTT	AGTGCGGCCGCCCCAGTCTCACAGGGGCCCAT
lnc-APUE-mut (site1)	GGACGCTGTTCGTGAAACTTACAGCAGCCCAGGAACCT	GTTTCACGAACAGCGTCCGTGGCTTACAGCAGCAGA
lnc-APUE-mut (site2)	ACGGAGGCTGAGGCACGAGGATTGCT	TCAGCCTCCGTTTCACGTGGGATT
E2F1-3'UTR	AGTCTCGAGAAGGGAAGGAGTCTGTGTGG	AGTGCGGCCGCAACCAAAGCAGGAGGGAACA

[a] the number in brackets indicates the location of primers on genes.
